# Production and First-in-Man Use of T Cells Engineered to Express a HSVTK-CD34 Sort-Suicide Gene

**DOI:** 10.1371/journal.pone.0077106

**Published:** 2013-10-21

**Authors:** Hong Zhan, Kimberly Gilmour, Lucas Chan, Farzin Farzaneh, Anne Marie McNicol, Jin-Hua Xu, Stuart Adams, Boris Fehse, Paul Veys, Adrian Thrasher, Hubert Gaspar, Waseem Qasim

**Affiliations:** 1 Molecular Immunology Unit, Institute of Child Health (ICH), University College London (UCL), London, United Kingdom; 2 Department of Haematological Medicine, The Rayne institute, Kings College London (KCL), London, United Kingdom; 3 Bone Marrow Transplant Unit, University of Hamburg, Hamburg, Germany; Karolinska Institutet, Sweden

## Abstract

Suicide gene modified donor T cells can improve immune reconstitution after allogeneic haematopoietic stem cell transplantation (SCT), but can be eliminated in the event of graft versus host disease (GVHD) through the administration of prodrug. Here we report the production and first-in-man use of mismatched donor T cells modified with a gamma-retroviral vector expressing a herpes simplex thymidine kinase (HSVTK):truncated CD34 (tCD34) suicide gene/magnetic selection marker protein. A stable packaging cell line was established to produce clinical grade vector pseudotyped with the Gibbon Ape Leukaemia Virus (GALV). T cells were transduced in a closed bag system following activation with anti-CD3/CD28 beads, and enriched on the basis of CD34 expression. Engineered cells were administered in two escalating doses to three children receiving T-depleted, CD34 stem cell selected, mismatched allogeneic grafts. All patients had pre-existing viral infections and received chemotherapy conditioning without serotherapy. In all three subjects cell therapy was tolerated without acute toxicity or the development of acute GVHD. Circulating gene modified T cells were detectable by flow cytometry and by molecular tracking in all three subjects. There was resolution of virus infections, concordant with detectable antigen-specific T cell responses and gene modified cells persisted for over 12 months. These findings highlight the suitability of tCD34 as a GMP compliant selection marker and demonstrate the feasibility, safety and immunological potential of HSVTK-tCD34 suicide gene modified donor T cells.

**Trial Registration:**

ClinicalTrials.gov NCT01204502 <NCT01204502>

## Introduction

Allogeneic haematopoietic stem cell transplants (SCT) from mismatched unrelated donors or haploidentical family donors are high risk procedures, requiring rigorous T cell depletion to mitigate against graft versus host disease (GVHD) [1]. Strategies to remove donor T cells include *in-vivo* antibody based depletion through the inclusion of serotherapy (for example Alemtuzumab, Antithymocyte Globulin, or OKT3) in the conditioning regimen or by *ex-vivo* depletion of T cells by magnetic bead based graft manipulation (for example, through enrichment of stem cells expressing CD34, or by depletion of T cells expressing CD3 or αβT cell receptors). Whilst stringently T-depleted grafts are less likely to cause GVHD, they also have reduced anti-viral properties and often lose graft versus leukaemia effects [2]. One approach designed to allow the infusion of mismatched donor T cells involves the stable introduction of a suicide gene to allow elimination of cells in the event of GVHD though the activation of specific prodrugs. The most extensively studied system uses gene modification with Herpes simplex thymdine kinase (HSVTK) which can activate Ganciclovir to induce cell death, and has now been tested in a number of clinical trials [3]–[10]. More recently a fusion gene encoding an inducible human caspase-9 apoptosis gene and modified human FK-binding protein has also been evaluated in pilot studies [11]. One prerequisite for this form of gene therapy, is the need to ensure that a very high proportion of infused cells encode the suicide gene, and thus all clinical trials to date have included linked selection marker genes. Bonini et al employed Neomycin based selection, subsequently switching to magnetic bead-antibody based selection of co-expressed truncated low affinity nerve growth factor receptor (ΔLNGFR) [3]. Alternatives include a truncated CD19 (ΔCD19) selection marker, used to enrich T cells expressing human caspase-9/FK-binding protein based suicide gene system [11]. Here we describe the first clinical data using a HSVTK suicide gene fused to a truncated splice variant of human CD34 (tCD34) [12]. Selection based on CD34 expression has an important advantage as it can be combined with Miltenyi CliniMacs reagents which are already widely used for CD34 stem cell selection. We, and others, have previously described pre-clinical variants of this system delivered by gamma-retroviral and HIV lentiviral vectors to human T cells [12]–[15]. Here we describe gamma-retroviral gene modification, enrichment and clinical use of human T cells expressing a modified HSVTK-CD34 sort-suicide fusion gene in three subjects following T cell depleted allogeneic SCT. This small study provides important proof-of-concept and safety data for the system.

## Materials, Methods and Subject Details

All subjects received treatment at Great Ormond Street Hospital, London under ethics approval from the UK Gene therapy advisory committee (GTAC) a national body overseeing ethical conduct of gene therapy studies. The study was regulated and monitored by the MHRA, UK. Parents provided written informed consent on behalf of all children. The protocol (see Protocol S1) for this study and supporting CONSORT checklist (see Checklist S1) are available as supporting information.

### 1. Plasmids and cell lines

**Figure 1:**
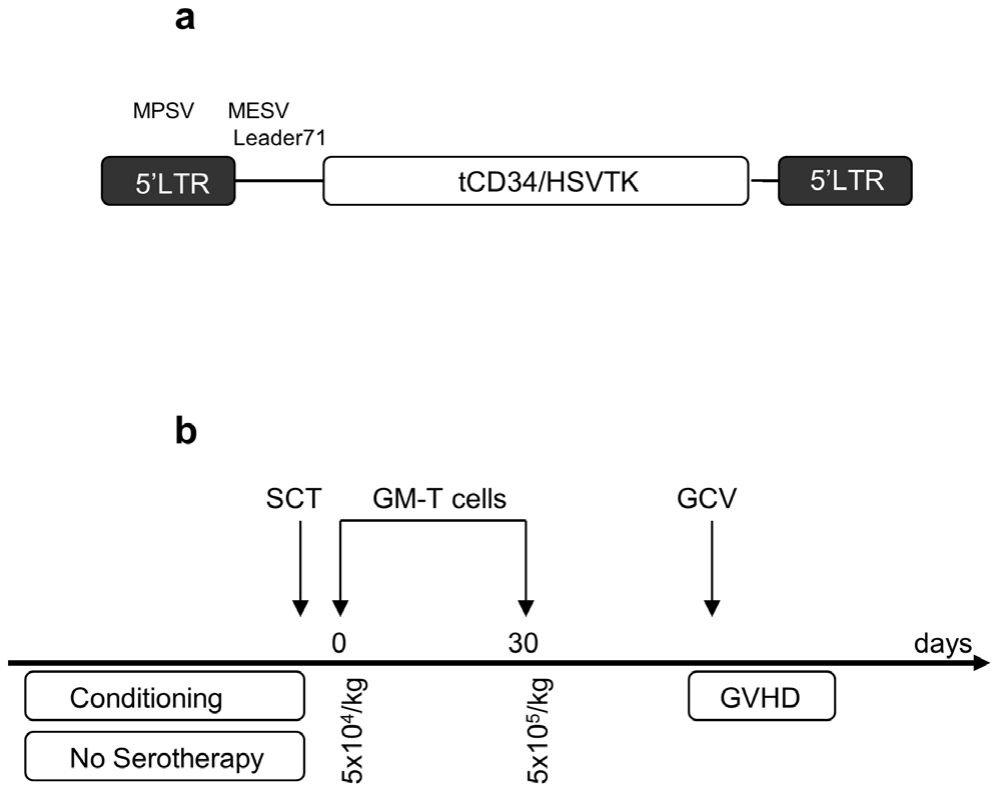
Vector configuration and study schema. 1a. A gamma retroviral platform incorporating long terminal repeals (LTRs) from Myeloproliferative sarcoma virus (MPSV) and leader sequence 71 derived from Murine embryonic stem cell virus (MESV). The splice site corrected herpes simplex virus thymidine kinase suicide gene (scHSVTK) fused to a truncated (splice variant) human CD34 gene is shown. 1b. Subjects undergoing CD34 selected mismatched allografts and receiving grafts carrying <5×10^4^ T cells/kg following conditioning (but not serotherapy) were eligible. Gene modified T cells were scheduled at two cell doses, the first 5×10^4^/kg the day following the stem cell graft, and the second programmed within 28 days at a higher dose of 5×10^5^/kg. In the event of GVHD>Grade I, Ganciclovir therapy was scheduled for seven days to eliminate gene modified T cells.

A gamma retroviral vector plasmid, encoding long terminal repeats from Myeloproliferative sarcoma virus (MPSV) and the leader 71 sequence from MESV and coding for a suicide/sort fusion gene comprising splice site corrected HSVTK fused to a truncated splice variant of human CD34 (Figure 1a) has been previously described [12] and was produced by Geneart (Germany) along with two independent accessory plasmids encoding ecotropic env and gag/pol, plasmids. Transiently produced ecotropic retroviral supernatant was produced in 293T cells (from a qualified master cell bank) and filtered (0.45 um) before transduction of PG13 cells (ATCC, CRL-10686), a stable packaging line producing Gibbon Ape Leukaemia Virus (GALV) pseudotyped retroviral vector [16]. A high titre clone was selected under GMP conditions by limiting dilution. Following production and characterisation of a master cell bank (Table 1), vector was produced in 10 layer HYPERFlasks (Corning, UK). Vector was harvested in X-Vivo 10, filtered (0.45 um) and cryopreserved in 100 ml bagged aliquots at −80C. Vector titres were estimated by flow cytometry for CD34 expression in HT1080 cells. End of production cells (EOP) and 5% of the vector harvest were subjected to release test analyses in accordance with harmonised European pharmacopeia guidelines by Bioreliance (Glasgow, Scotland) or at the Institute of Child Health, London (Table 1).

**Table 1:** Release characterisation of retroviral stocks.

Investigation	Test Article	Method	Specification
*Replication competent retroviruses (RCR)	EOP cells	PG4 S+L- Assay	No CPE detected
**Titre of the produced supernatant	Vector supernatant	Flow analysis	>10^5^ infectious particles/ml detected on basis of CD34 expression
**Transgene functionality	Vector supernatant	MTT assay	Survival of T cells transduced with HSVTK <20% at concentrations of GCV at 10 µM, and absence of viable cells detected on trypan blue staining
*Sterility of the cells	Vector supernatant	BacTec	No microbial growth detected
**Replication competent retroviruses (RCR)	Vector supernatant	PG4 S+L- Assay	No CPE detected
*Endotoxin	Vector supernatant	Chromogenic kinetic LAL test	<5 EU/ml.
*Mycoplasma by indicator cell culture	Vector supernatant	Culture and Immunofluorescence	No mycoplasma detected
*Adventitious pathogens	Vector superntatant	Inoculation of adult and suckling mice, and guinea pigs	No viral contamination detected

* Undertaken by Bioreliance (Glasgow, Scotland) under harmonised European pharmacopeia.

** Institute of Child Health, London. A similar schedule of characterisation was applied to the PG13 Master cell bank.

### 2. T cell transduction & selection

Allogeneic donors had completed prior virological screening for peripheral blood SCT. Donor lymphocytes were subsequently obtained from ficolled whole blood (P1) or non-mobilised leukapheresis collection (P2, 3). Cells were re-suspended in gas permeable 100 ml cell differentiation bags (Miltenyi biotech, Germany) at 10^6^/ml in X-Vivo 10 (Lonza, Belgium) supplemented with 5% human AB serum (Lonza, USA) and 100 u/ml of human recombinant interleukin 2 (Proleukin, Novartis, USA,) and activated with Dynabeads® ClinExVivo™ CD3/CD28 (Invitrogen, UK) at a ratio of 1∶1. Cell density was maintained in the range of 0.5–1.0×10^6^/ml throughout with additional IL2 supplementation very 48 hrs. Two rounds of vector exposure were undertaken after 48 and 72 hours with CH-296 coated bags (RetroNectin, Takara bio Inc, Japan), preloaded with retrovirus by centrifugation. Following semi-automated magnetic bead removal using a Dynal ClinExVivo MPC (Invitrogen, UK) cells were rested overnight before using CliniMacs CD34 selection kit (Miltenyi biotech, Germany) to select CD34 expressing transduced T cells. Transduction efficiency and purification were assessed using mouse anti-human CD34 PE conjugated mAb (BD Biosciences, Europe) stained and analysed using flow cytometry (BD Biosciences), Cells were again rested overnight and then cryopreserved in dose aliquots of 5×10^4^/kg and 5×10^5^/kg. Reagents are detailed in Table 2 and the transduction procedures provided in full in Table 3.

**Table 2:** GMP T cell transduction reagents.

Reagents	Cat no/Lot no	Company
X VIVO 10	8SP200	Lonza, Belgium
L-glutamine	17-905C	Lonza, USA
Human AB serum	14-498E	Lonza, USA
Recombinant Human Interleukin-2 (IL-2) [Proleukin]	00101/0936	Novartis, USA; Procured via Great Ormond Street Pharmacy
CD3 and CD28 Beads [Dynabeads® ClinExVivo™ CD3/CD28]	402.03D	Invitrogen, Norway
GMP-grade CH-296 (RetroNectin)	T100B	Takara Bio Inc, Japan
CliniMacs CD34 selection kit	171-01	Miltentyi Biotech, Germany
CliniMACS TUBING SET	161-01	Miltentyi Biotech, Germany
100 ml cell differentiation Bags	170-076-400	Miltentyi Biotech, Germany
Phosphate Buffer Saline/EDTA	700-25	Miltentyi Biotech, Germany

**Table 3:** GMP compliant T cell transduction procedure.

Day 1 Activation	1.Resuspend cells at 1×10^6^/ml in multiple 100 ml Miltenyi bags; 2.Coat 2× number of T cell bags with retronectin (1 mg/ml in 10 ml PBS)
Day 3 Transduction Round 1	1.Thaw vector; 2.Remove RN from bags and add 50 ml vector per bag; 3.Spin bags at 1000 g, 40 min; 4.Transfer cell suspension to each bag (1∶1 ratio)
Day 4 Transduction Round 2	1.Thaw vector; 2. Remove RN from bags and add vector; 3. Spin bags at 1000 g, 40 min; 4. Volume reduce; 5. Add IL2 to final concentration 100 u/ml
Day 6 Culture	Add IL2 to final concentration 100 u/ml
Day 7 Bead removal	1.Assess CD34 expression by flow cytometry; 2 Remove CD3/CD28 beads using MagSep (Dynal); 3.Rest overnight in X-Vivo 10+5% AB serum+IL2 100 u/ml
Day 8 Positive selection	1.CliniMacs selection of CD34+ T cells; 2.Rest overnight in X-Vivo 10+5% AB serum+IL2 100 u/ml
Day 9 Dose preparation	1.Flow cytometry for CD34 purity; 2.Phenotype analysis by flow cytomtetry; 3.Archive samples for RCR testing; 4.Cryopreserve cells in dose aliquots

### 3. Assessment of sensitivity to the prodrug Ganciclovir

Transduced T cells were exposed to 10 uM Ganciclovir (GCV, Roche Limited, UK) and after 72 hours viability was assessed in triplicate by spectrophotometry using a 3-(4,5-dimethylthiazol-2-yl)-2,5-diphenyltetrazolium bromide assay (MTT, Sigma, USA) as previously described [17]. The assay measures mitochondrial activity and thus background levels of up to 20% were detectable even when no cells were sufficiently viable to mediate trypan blue exclusion.

### 4. Proliferation and alloreactivity responses

To assess alloreactivity T cells and irradiated (30 Gy) stimulator cells were suspended at 10^6^/ml in X-vivo 10/5% AB serum and stimulator cells and 100 ul of each plated in relevant autologous∶allogeneic combinations, in triplicate in 96-U well plates. After a 5 day culture, cells were pulsed with 0.5 µCi/well ^3^H-thymidine (Amersham Bioscience) for 16 hours and were then harvested onto a filtermat using a Wallac 96 well plate harvester. Radioactive incorporation was measured using a Wallac counter. Responses to polyclonal stimulation by anti-human CD3 (OKT3, Ebioscience, UK) were also assessed in the presence or absence of 10 uM GCV.

### 5. Transfer and tracking of T cell mediated virus specific immunity

Virus specific responses were assessed in subjects and donors (where possible) using an IFN-γ secretion assay-detection kit (PE) (Miltenyi Biotech, Germany). Following overnight stimulation with relevant antigens adenovirus hexon (ADV, Miltenyi Biotec, Germany), Varicella zoster (VZV) and H1N1 (National Institute for Biological Standards and Control (NIBSC), UK) or staphylococcal enterotoxin B (SEB, Sigma, UK), IFN-γ secreting cells were labelled using a bi-specific monoclonal antibody, specific for both IFN-γ and CD45. Cells were stained in RPMI/1% AB serum and cultured at 37°C for 45 minutes. The IFN-γ positive secreting cells were labelled using IFN-γ PE detection Ab, anti-CD8FITC mAb (Becton Dickinson,USA), and APC-labelled CD4 mAb (Becton Dickinson, USA), and then analyzed by flow cytometry.

### 6. Regulatory Approvals, patient characteristics and procedures

All subjects were treated under approvals secured from the UK Medicine and Healthcare Products Regulatory Agency (MHRA) and Gene therapy advisory committee (GTAC). P2 and P3 were treated as part of a registered clinical trial (NCT01204502) and P1 treated following approval from both MHRA and GTAC.

All three subjects received grafts comprising CD34 selected peripheral blood stem cells (PBSC) following chemotherapy conditioning without serotherapy, and received an initial dose of 5×10^4^/kg HSVTK-CD34 modified T cells, within one day of stem cell grafting. All received prophylaxis against GVHD with Cyclosporin in combination with Mycophenolate Mofetil (MMF). P1, a child with Fanconi anaemia, was the recipient of a second mismatched unrelated donor (MMUD) graft following relapse of MDS following an initial reduced intensity procedure. P2 and P3 were infants undergoing paternal haploidentical (haplo) PBSCT to treat severe combined immunodeficiencies (SCID) and had pre-existing viral complications with H1N1 influenza (P2) and Adenovirus (P3).

### Statistical analysis

Where indicated, student t tests were applied to cell proliferation and survival data.

## Results and Discussion

### Production of HSVTK-CD34 gene modified T cells

**Figure 2:**
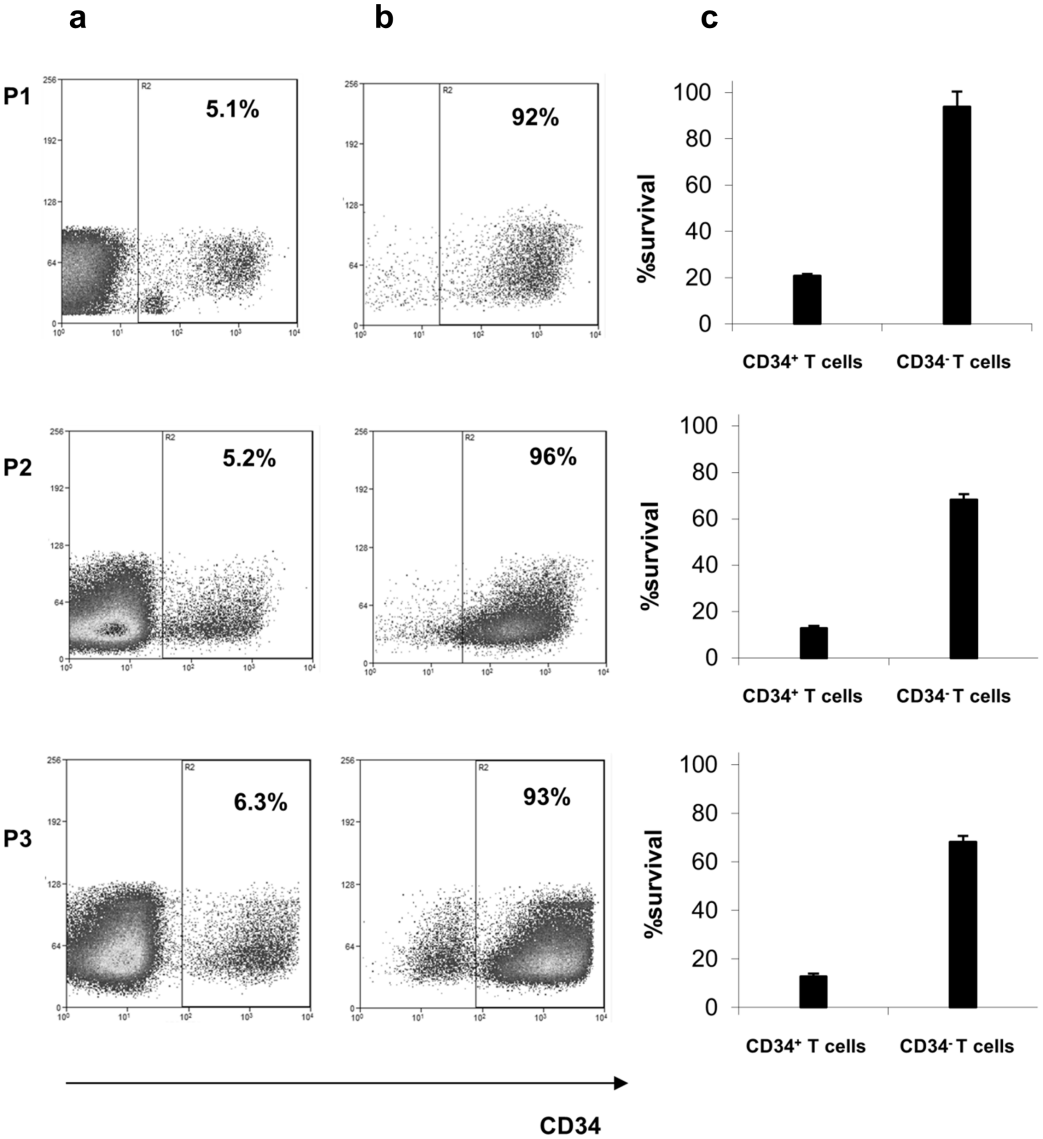
Transduction, enrichment and suicide gene function. (a) Flow cytometry of peripheral blood lymphocytes after transduction. Cells were activated with anti-CD3/28 beads and underwent two rounds of exposure to vector before removal of activation beads and magnetic bead enrichment using a CliniMacs device. (b) Transduced T cells were enriched (CD34+) to >90% purity for all three products. (c) Upon exposure to the prodrug Ganciclovir (GCV, 10 uM), engineered cells from all three donors had reduced survival compared to non-modified controls (P<0.001). Means of triplicate wells and standard error of means are shown.

**Figure 3:**
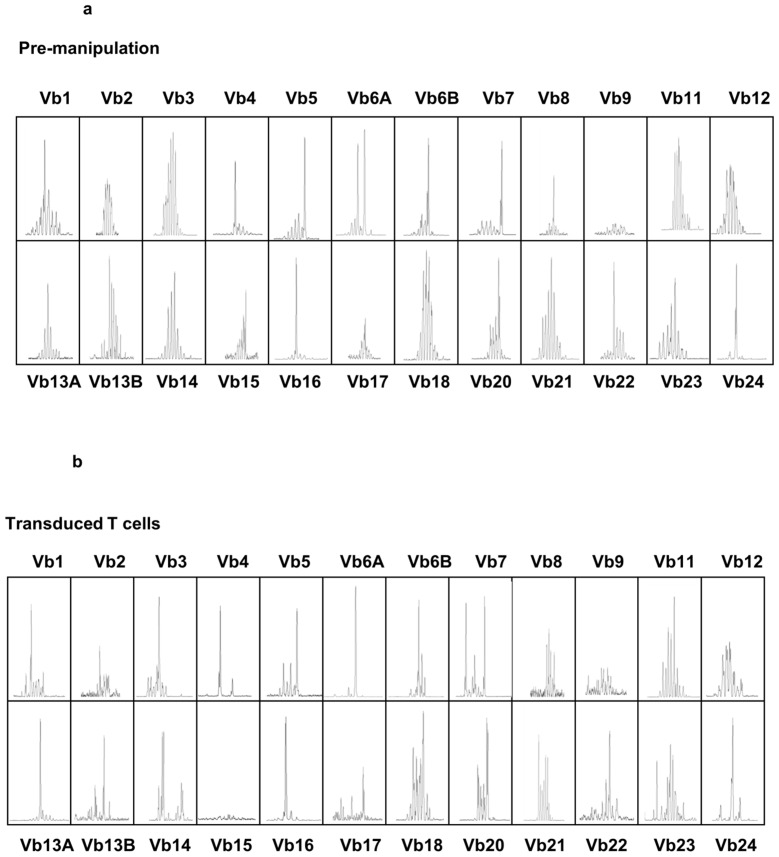
T cell repertoire diversity before and after modification. Complementarity determining region-3 (CDR3) T-cell receptor (TCR) spectratyping was performed as previously described [18]. Briefly, RNA was extracted and cDNA prepared from pre- and post-transduced cells. Twenty four Vβ-specific primers were used with a fluorescent-labelled constant region (Cβ)-specific primer to RT-PCR amplify the CDR3 region of the TCR β chain. Products were run on an AB3130 Genetic Analyzer and analysed using GeneMapper v4.0 software (Applied Biosystems, Warrington, UK). Representative data for P2 is showing preservation Vβ family distributions is shown.

**Figure 4:**
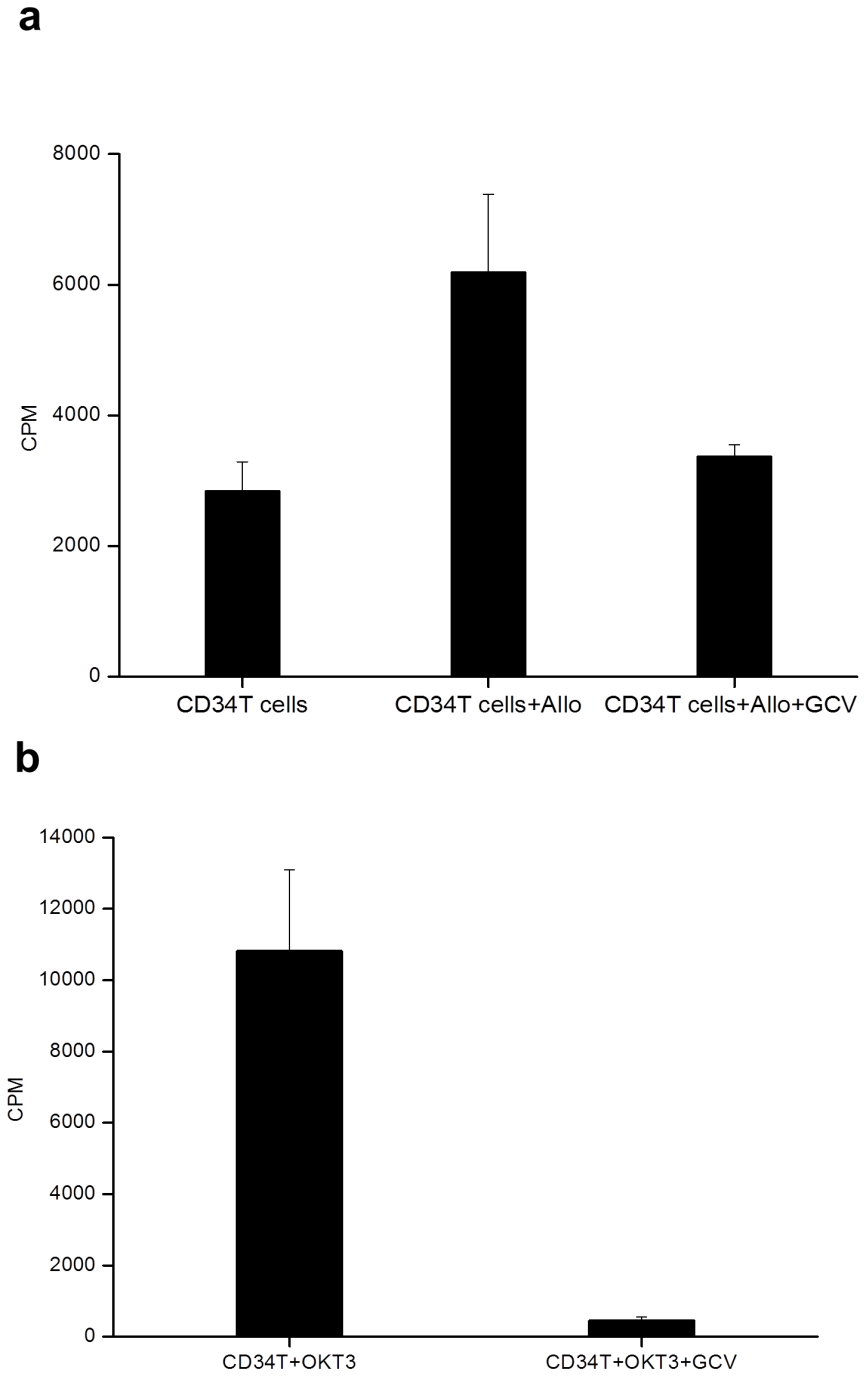
Proliferation and alloreactivity responses. In the upper panel, CD34TK modified T cells were co-cultured with irradiated allogeneic peripheral blood monuclear stimulator cells and proliferation was measured by ^3^H-thymidine incorporation. Cells mounted significant responses against allogeneic target cells (p = 0.02) whereas in the presence of 10 uM GCV, proliferation was significantly reduced (p = 0.05). In the lower panel, proliferation of gene modified T cell responses following polyclonal stimulation by anti-human CD3 were abrogated in the presence of 10 uM GCV (P<0.01). Means of triplicate wells with standard error of mean are shown.

A replication defective gamma retroviral vector suitable for T cell modification, with intact long terminal repeat promoter elements, and encoding a splice site corrected HSVTK-tCD34 fusion gene was produced under GMP conditions using a stable producer clone expressing accessory packaging genes and the Gibbon Ape Leukaemia Virus envelope (see M&M). Previous clinical trials have used vectors encoding HSVTK linked to neomycin resistance and/or ΔLNGFR [3], [4], [8]. We used an abbreviated transduction and selection process lasting 8 days, employing anti-CD3/CD28 activation beads and animal serum-free conditions, which preserved T cell repertoire diversity and retained T cell alloreactivity and antiviral function as previously described [18]. The entire process, including CliniMacs based CD34 selection of engineered T cells was performed using closed bag systems under GMP compliant conditions. Details of transduction efficiency, magnetic bead mediated enrichment and cell yields are provided in Table 4. In all three cases, between 5–6% of donor T cells were modified (Figure 2a) ensuring low vector copy numbers and these cells were then enriched on the basis of CD34 expression to 92–96% purity to produce target cell doses for infusion (Figure 2b). Flow cytometry confirmed that the majority of cells (88–99%) were CD3^+^ T cells, with the expected sensitivity to GCV *in vitro* (Figure 2c). T cell receptor repertoires were Gaussian with all families represented (Figure 3). Modified T cells exhibited GCV sensitive anti-CD3 mediated proliferation and alloreactivity against allogeneic irradiated peripheral blood mononuclear cells (Figure 4).

**Table 4:** Production of donor HSVTK-CD34 T cells.

Patients	P1	P2	P3
Donor type	MMUD	Haplo	Haplo
CD3 after transduction	99%	97%	88%
CD3^+^CD4^+^	78%	28%	49%
CD3^+^CD8^+^	21%	65%	50%
Transduction efficiency	5.1%	5.2%	6.3%
Purification	92%	96%	93%
Viability	96%	92%	93%
Transduced T cell number	31×10^6^	57×10^6^	190×10^6^
% survival in 10 uM GCV	20%	13%	11%
Dose1 (<5×10^4^/kg)	1.8×10^6^	2.5×10^5^	3.4×10^5^
Dose2 (<5×10^5^/kg)	17.5×10^6^	5.0×10^6^	Not given

### First-in-man use of HSVTK-CD34 T cells

**Figure 5:**
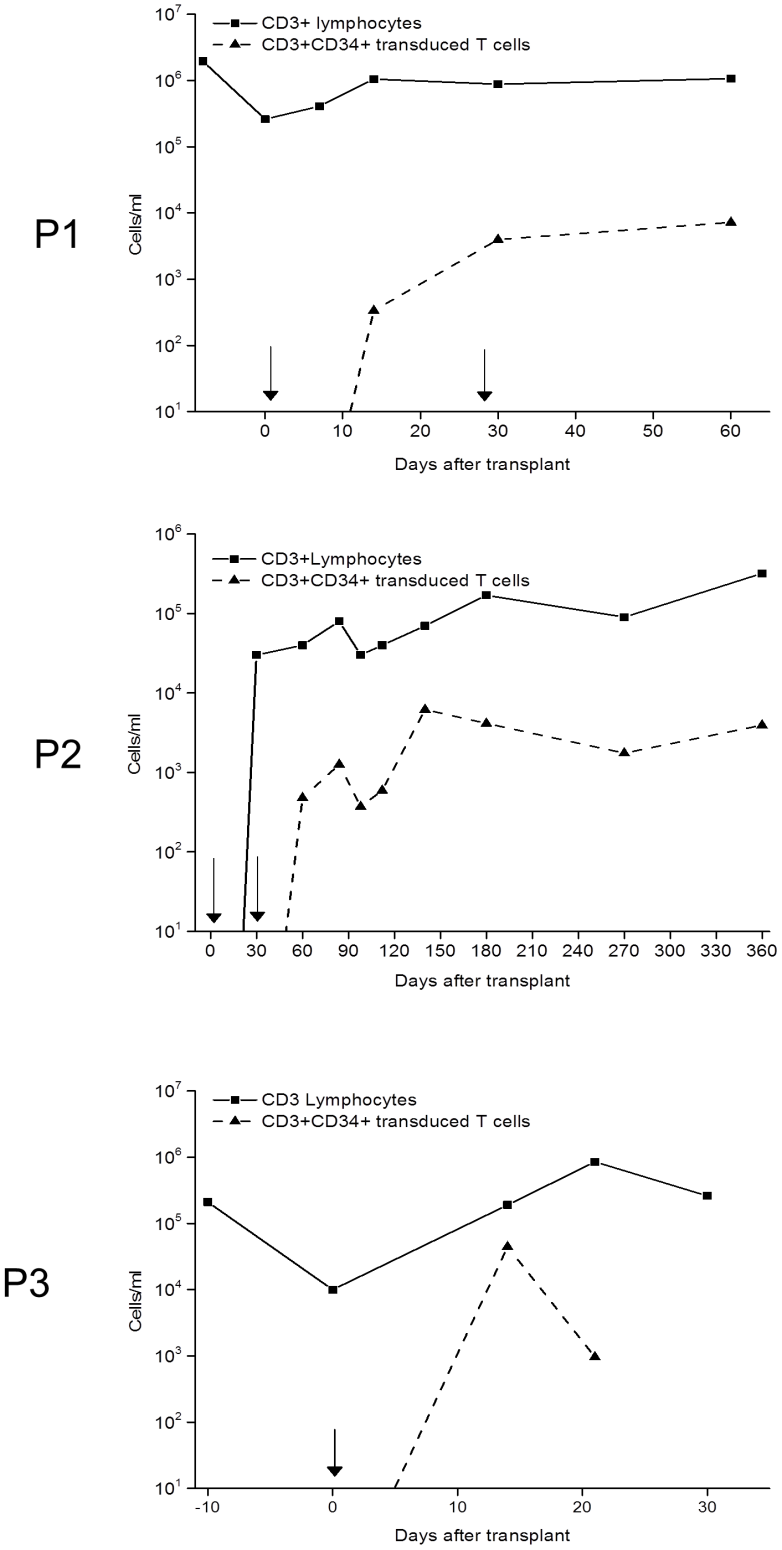
T cell reconstitution in patients after cell therapy. P1, a child with Fanconi anaemia, underwent a second mismatched donor, CD34 selected stem cell graft after in the context of relapsed MDS. Donor HSVTK/CD34 modified T cells were infused in two dose aliquots and were detectable at low level in peripheral blood for over 12 weeks before the patient died of disease relapse. The persistence of non-modified T cells reflects the reduced intensity conditioning and absence of serotherapy. P2, an infant with RAG1 deficient SCID had no pre-existing T cell immunity and was conditioned whist infected with H1N1 influenza. Modified T cells persisted for over 12 months, with eventual recovery of thymic derived donor T cells after one year and normalisation of immunity. P3 suffered Ligase IV deficiency, a form of radiosensitive SCID. Expansion of modified donor T cells was detected within two weeks of first infusion, but the patient died from mucositis related pulmonary and gastrointestinal haemorrhage before dose escalation.

**Figure 6:**
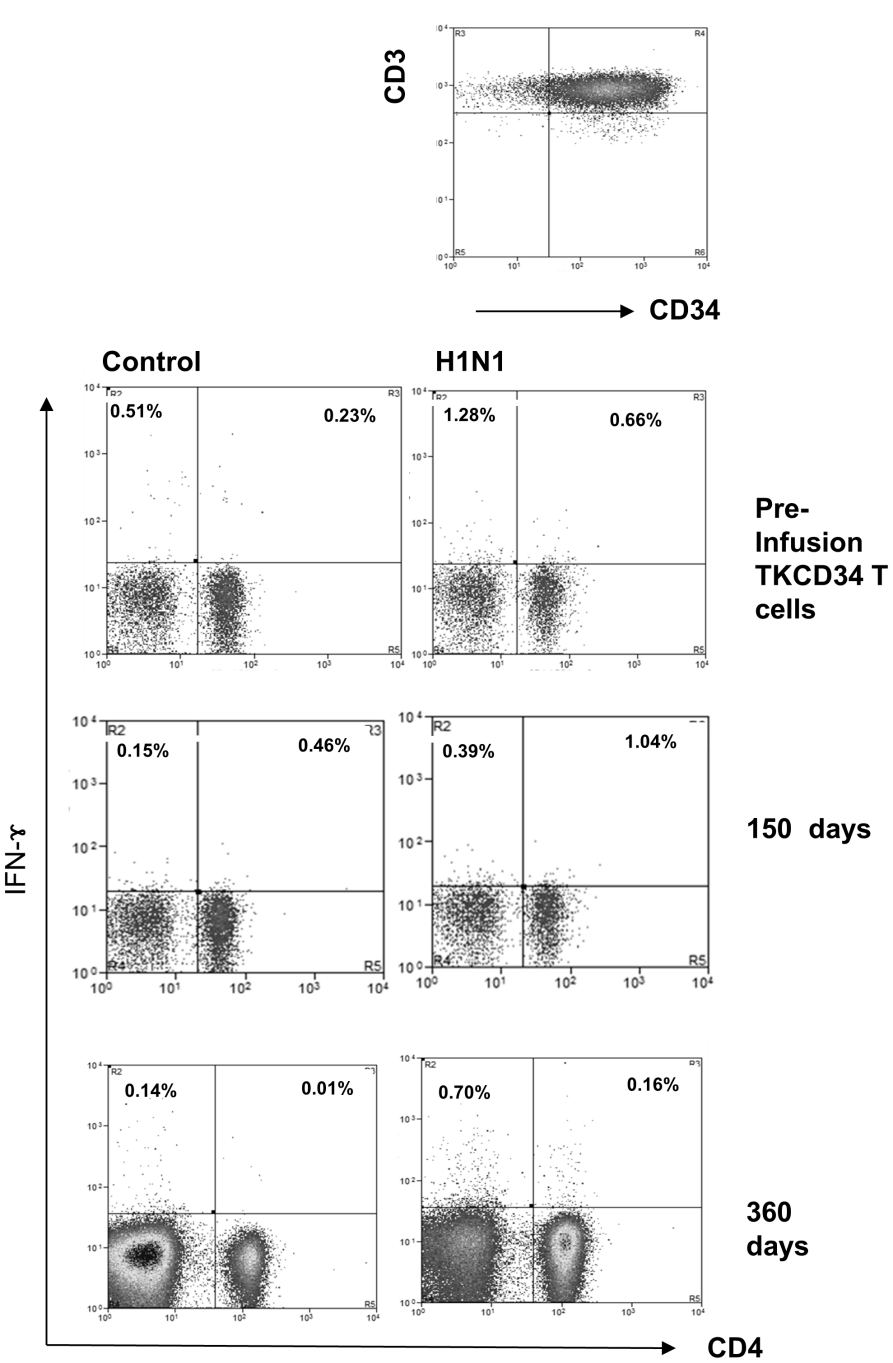
Transfer and tracking of T cell mediated virus specific immunity. Most compelling, and beneficial, was transfer of immunity against pandemic H1N1 infulenza in P2. The haploidentical donor had been electively vaccinated against the strain before leukapheresis harvest of peripheral blood lymphocytes. The transduced and CD34 enriched populations exhibited specific IFNγ responses against HI1N1 compared to non-stimulated control cells. Samples collected 150 days after donor lymphocyte infusion from the patient showed similar H1N1 specific IFNγ responses, which coincided with clearance of persistent H1N1 respiratory infection. These responses were still detectable after 350 days.

All subjects underwent CD34 selected mismatched stem cell grafting after conditioning which did not include any form of serotherapy. Infusions of HSVTKCD34 T cells (5×10^4^/kg) were tolerated in all subjects without any acute toxicities (Figure 5). Only P1 developed skin GVHD (Grade I) and this was self-limiting, not requiring additional systemic therapy. Of note, the study protocol, under the direction of UK regulatory authorities, limited the maximum dose of donor T cells to 5×10^5^/kg, a dose not expected to cause significant GVHD and lower than that used in previous trials where GVHD was encountered more frequently. Unfortunately, P1 was transplanted in the presence of relapsed MDS, and remission was not achieved after escalated dose cell therapy (5×10^5^/kg). However, reactivation of localized varicella zoster infection was rapidly controlled and T cell responses against VZV antigen were detectable in peripheral blood using an interferon gamma capture assay (not shown). P2 (RAG1 deficient SCID) was transplanted with pre-existing H1N1 influenza colonisation which was resistant to Oseltamavir and was requiring ongoing Zanamavir therapy. The halpoidentical donor was vaccinated against influenza, including the 2009 pandemic H1N1 strain and specific interferon-γ based responses were detectable in the enriched gene modified T cell donation (Figure 6). The subject received both programmed cell doses without developing GVHD and over the following months eradicated H1N1 influenza. Specific responses were detectable in peripheral blood for over 12 months (Figure 6), although cellular immune reconstitution was slow and disrupted by autoimmune haemolysis requiring systemic therapy with corticosteroids and Rituximab. Nonetheless, by twelve months after transplantation, there was robust T cell recovery mediated by *de novo* thymopoieisis. Persistence of HSVTKCD34 T cells was documented throughout this period and interestingly recent post pandemic studies of T cell responses against H1N1 have reported similar interferon mediated T cell responses in transplant and vaccinated hosts [19]. P3 suffered from a radiation sensitivity disorder (Ligase IV deficiency) with pre-existing infectious complications including pneumocystis carinii, rhinovirus, enterovirus and adenovirus. The patient developed significant early mucositis following conditioning. There was a notable but transient rise in peripheral blood T cells within two weeks of SCT but no evidence of GVHD. Virus specific responses against adenovirus hexon antigen were detectable in the donor and the child after cell infusion (not shown). However, the underlying radiosenstivity disorder probably predisposed to severe the mucositis following conditioning, and subsequent catastrophic gastrointestinal and pulmonary haemorrhage occurred before the second dose of cell therapy, and in absence of apparent GVHD.

We were able to demonstrate T cell mediated, antigen specific responses against reactivating viruses, Varicella zoster (P1), H1N1 (P2) and Adenovirus (P3). These viruses are often problematic after SCT and whilst they can often be partially controlled with antiviral drugs, require intact cellular immunity for clearance [20]. The beneficial antiviral effects may have been mediated by both engineered and non-modified T cell populations, but unfortunately because of the low frequency of detectable virus specific populations and low lymphocyte counts in peripheral blood following SCT, it was not possible to characterise effector cells in detail. However, clearance of similar viral pathogens after T cell depleted transplants generally requires several months of immune recovery. We found that there was delay of almost 12 months in P2 until significant thymic mediated T cell reconstitution occurred, and in the interim he presumably benefited from adoptive transfer of anti-H1N1 immunity from the donor. Furthermore, it has been reported adult patients receiving TK modified T cells experience greater than expected levels of thymopoiesis and it has been postulated that this may be mediated by increased levels of IL-7 following donor lymphocyte infusion [21]

Whilst the HSVTK element in our vector is potentially immunogenic, responses were not anticipated in the immuodeficient subjects treated here. Previous trials using suicide gene modified T cells documented clearance of engineered T cells in immunocompetent subjects who mounted immune responses against HSVTK or NeoR antigens [22]. Alternative non-immunogenic humanised suicide genes are being developed, most notably inducible variants of caspase genes, which have recently been used in pilot studies, although access to the dimerising agent required to elicit cell death is restricted [11].

In summary we have demonstrated under GMP conditions the feasibility of T cell modification and selection using a HSVTK-tCD34 suicide/selection system, and provide first-in-man data for this approach in the setting of mismatched allogeneic SCT. Whilst GVHD was not problematic at the cell doses administered, recovery of specific antiviral immunity was demonstrated in all three subjects. The reagents and procedures are readily adaptable for other gene based therapies, including emerging anti-tumour and anti-viral cellular therapies.

## Supporting Information

Protocol S1 Trial Protocol.
(PDF)

Checklist S1 CONSORT Checklist.
(PDF)
