# Supplementary material for: Production and First-in-Man Use of T Cells Engineered to Express a HSVTK-CD34 Sort-Suicide Gene
Source: PLoS One. 2013 Oct 21;8(10):e77106. doi: 10.1371/journal.pone.0077106 (PMC3804528; doi:10.1371/journal.pone.0077106)
Supplement: Protocol S1 — Trial Protocol. (PDF) [file pone.0077106.s001.pdf]

UCL INSTITUTE OF CHILD HEALTH

CENTRE FOR IMMUNODEFICIENCY

Great Ormond Street Hospital for Children NHS Trust

Title: Phase I/II Clinical Trial of T Cell Suicide Gene Therapy Following Haploidentical Stem Cell Transplantation

CONFIDENTIAL

EudraCT: 2005-001925-27

Sponsor Protocol Number: 06-MI-04

Protocol Version: 8, dated 17th November 2011

TRIAL CONTACTS

CHIEF INVESTIGATOR

Dr Waseem Qasim
Consultant in Immunology
Molecular Immunology Unit
Institute of Child Health
30 Guilford Street
London WC1N 1EH
Tel: 020 7905 2270 Fax: 020 7905 2810
Email: W.Qasim@ich.ucl.ac.uk

PRINCIPAL INVESTIGATORS

Dr Bobby Gaspar
Molecular Immunology Unit
Institute of Child Health
30 Guilford Street
London WC1N 1EH
Tel: 020 7905 2319 Fax: 020 7905 2810
Email: h.gaspar@ich.ucl.ac.uk

Professor Adrian Thrasher
Molecular Immunology Unit
Institute of Child Health
30 Guilford Street
London WC1N 1EH
Tel: 020 7905 2660 Fax: 020 7905 2810
Email: a.thrasher@ich.ucl.ac.uk

Dr Paul Veys
Bone Marrow Transplant Unit
Great Ormond Street Hospital for Children NHS Trust
London WC1N 3JH
Tel: 020 7813 8434 Fax: 020 7813 8552
Email: VeysP@gosh.nhs.uk

SPONSOR

Great Ormond Street Hospital for Children NHS Trust
Great Ormond Street
London, WC1N 3JH

Sponsor contact:
Biren Patel
Clinical Trials Coordinator
Joint Research & Development Office
Institute of Child Health
30 Guilford Street
London WC1N 1EH
Email: biren.patel@gosh.nhs.uk
Tel: 020 7905 2863
Fax: 020 7905 2201

LABORATORIES

Gene Therapy Laboratory (GOSH)

Hong Zhan
Gene Therapy Clinical Scientist
Institute of Child Health
30 Guilford Street
London WC1N 1EH
E-mail: h.zhan@ich.ucl.ac.uk

Kathryn Parsley
Gene Therapy Clinical Scientist
Molecular Immunology Unit
Institute of Child Health
30 Guilford Street
London WC1N 1EH
k.parsley@ich.ucl.ac.uk

Dr Kimberly Gilmour
Clinical Scientist
Great Ormond Street Hospital for Children NHS Trust
London WC1N 3JH
Tel: 020 74059200
Email: GilmoK@gosh.nhs.uk

Clinical Laboratories

GOSH pathology laboratories (Immunology/Haematology/Biochemistry/
Chemical Pathology/Microbiology)
Great Ormond Street

Signature Page

The trial will be conducted in compliance with the protocol, GCP and the applicable regulatory requirement(s).

Chief Study Investigator:

Name:

Signature:

Date:

On behalf of the Sponsor,
Great Ormond Street Hospital for Children NHS Trust:

Name:

Signature:

Position:

Date:

Protocol Synopsis

**Title**	Phase I/II Clinical Trial of T Cell Suicide Gene Therapy Following Haploidentical Stem Cell Transplantation
**Objectives**	1. Generation of genetically modified (GM) donor T cells with a retroviral vector encoding a suicide gene/selection marker fusion gene<br>2. Administration of GM donor T cells to facilitate immune reconstitution following haploidentical HSCT<br>3. Evaluation of T cell engraftment kinetics and stability<br>4. Elimination of GM-T cells using Ganciclovir in the event of GVHD<br>5. Longitudinal evaluation of clinical effect in terms of immune reconstitution, leukaemic relapse, and survival
**Design**	Non-randomised open label Phase I/II clinical study of HSVTK transduced donor T lymphocyte infusions in the haploidentical setting.
**Inclusion Criteria**	1. Patients with primary immunodeficiencies, haematological malignancies or metabolic disorders at GOSH (children of both sexes, aged 0 to 16 years) undergoing haploidentical transplant<br>2. Both patient and donor must give informed consent in writing.<br>3. The donor must be willing, able and available for donation of T cells by collection of whole blood or leukapheresis.<br>4. The patient should be free of serious intercurrent illness.
**Exclusion Criteria**	1. Donor unfit or unavailable<br>2. Donor positive for Hepatitis B or C, or HTLV-1, or HIV<br>3. Patient receiving Ganciclovir, Aciclovir, Cidofovir a result of active CMV, adenovirus, varicella zoster or herpes simplex infection infection<br>4. GVHD $\geq$ grade II before infusion of GM- T cells (see 6.6)<br>5. Serious intercurrent illness
**Dose / Regimen Trial Medication**	GM-T cells will be given at 1 month intervals, providing that there is no significant GVHD<br>• DOSE 1 <$5x10^4$ cells/kg<br>• DOSE 2 <$5x10^5$ cells/kg
**Treatment duration**	The transduced T cells are administered intravenously over 5-10 minutes (entire contents to be administered)
**Number patients**	We aim to treat 5-10 patients
**Efficacy Assessments**	1. Detection of GM-T cells (flow cytometry, quantitative PCR for HSVTK if transduced cells <1% on cytometry)<br>2. Absolute CD4 count (Flow cytometry)<br>3. T cell repertoire analysis (TCR spectratype) and thymic output (TRECs)<br>4. T cell mitogen responses (PHA)<br>5. Production of endogenous antibody (IgM) ans tetanus specific vaccination responses (if reconstituted and vaccinated at d +170)<br>6. Minumal residual disease (by PCR in the case of malignancy and where a marker is routinely available)<br>7. Donor chimerism
**Safety Assessments**	1. Evaluation of Adverse reactions (AR/SSAR/SUSAR) and Serious Adverse Events (SAE)<br>2. Replication Competent Retrovirus Testing RCR (Blood, Bone marrow)<br>3. Routine monitoting to include FBC, U+Es, LFTs
**Trial duration**	Children undergoing HSCT routinely undergo regular monitoring into adult hood. For the majority of patients the period of followup will be 2 years post administration of the transduced T cells
**End of Study**	The end of the study is defined as the last patient's last scheduled visit according to the protocol, which will be the 12 month followup of the last patient entered into the trial.

CONTENTS

TRIAL CONTACTS....2

ABBREVIATIONS USED 8

1. LAY SUMMARY....9

2. OBJECTIVES OF THE STUDY....10

3. ROLE OF T CELLS IN HSCT....11

3.1. GVHD FOLLOWING ALLOGENEIC HSCT....11
3.1.1 DEFINITION 11
3.1.2 PATHOGENESIS....11
3.1.3 RISK FACTORS FOR GVHD....11
3.2 MANAGEMENT OF GVHD....12
3.3 DEPLETION OF DONOR T CELLS FROM ALLOGENEIC GRAFTS 12
3.3.1 GRAFT FAILURE FOLLOWING T CELL DEPLETION....13
3.3.2. DELAYED IMMUNE RECONSTITUTION 13
3.3.3 RELAPSE OF LEUKAEMIA....14
3.4 DONOR LYMPHOCYTE INFUSIONS 14

4. T CELL SUICIDE GENE THERAPY....16

4.1. THE HSVTK/GCV SUICIDE GENE SYSTEM....16
4.2. RETROVIRAL VECTORS....16
4.3. PG13 PACKAGING CELL LINE 18
4.4 PROCEDURES FOR THE TRANSDUCTION OF T CELLS....18
4.5 ANIMAL STUDIES....19
4.6 HUMAN CLINICAL TRIALS OF T CELL SUICIDE GENE THERAPY....19
4.7 IMMUNE RESPONSE TO HSVTK 21
4.8 GANCICLOVIR RESISTANCE....21
4.9 FUNCTIONAL POTENTIAL OF TRANSDUCED T CELLS 21

5. PROCEDURE FOR THE PREPARATION OF GM-T CELLS....24

5.1 CONSTRUCTION OF RETROVIRAL VECTOR 24
5.2. PRODUCTION OF GALV PSEUDOTYPED VIRUS 24
5.3. T CELL TRANSDUCTION....24
5.4. EFFICIENCY OF CD34 SELECTION....24
5.5. MOLECULAR ANALYSIS OF TRANSDUCED CELLS 24
5.6. FUNCTION OF SUICIDE GENE....25
5.7 IMP ACCOUNTABILITY AND TRACEABILITY….25

6. HSVTK TRANSDUCED DONOR T LYMPHOCYTE INFUSIONS FOLLOWING HAPLO-IDENTICAL TRANSPLANTAION 26

6.1. RATIONALE....26
6.2. STATEMENT OF AIMS 26
6.3 PATIENT POPULATION 26
6.3.1 26
INCLUSION CRITERIA (AS FOR HSCT) 26
6.3.2 EXCLUSION CRITERIA....27
6.4. CONDITIONING REGIMES 27
6.5 HARVEST OF DONOR T CELLS....27
6.6.1 TIME OF GM-T CELL INFUSION 27
6.6.2 DOSE SCHEDULE AND ADMINISTRATION OF GM-T CELLS....28
6.7 TREATMENT OF SUBSEQUENT CMV INFECTION 28
6.8 GVHD....28
6.8.1 GVHD PROPHYLAXIS....29
6.8.2 TREATMENT OF GVHD....29
6.9 CLINICAL CARE 29
6.10 LABORATORY FOLLOW-UP 29
6.10.1 ROUTINE MONITORING …… 29
6.10.2 TRACKING T CELLS 29
6.11 OUTCOME MEASURES....30
6.12. WITHDRAWAL/REPLECEMENT OF INDIVIDUAL SUBJECTS....30

6.13. OFF STUDY CRITERIA 30
6.14 FOLLOW-UP OF WITHDRAWN SUBJECTS....30
6.15. STATISTICAL CONSIDERATIONS....31

7. SAFETY....32

7.1 RISK TO THE DONOR....32
7.1.1 LEUKAPHAESIS/BLOOD DONATION 32
7.2 RISKS TO THE RECIPIENT 32
7.2.1 RISKS OF INSERTIONAL MUTAGENESIS 32
7.2.2 RISKS OF USING A TRUNCATED CD34 SELECTION MARKER....32
7.2.3 GERMLINE TRANSMISSION OF VECTOR SEQUENCES....32
7.2.4 QUALITY CONTROL OF HARVEST AND TRANSDUCTION PROCESS 33
7.2.5 HANDLING OF TRANSDUCED CELLS....33
7.2.6 LACK OF GM-T CELL EFFICACY....33
7.2.7 FAILURE OF HSVTK/GCV SYSTEM 33
7.2.8 UNEXPECTED EVENTS OR OUTCOMES....34
7.3. PUBLIC HEALTH CONSIDERATIONS....34
7.4. GOOD CLINICAL PRACTICE & AND ETHICAL ISSUES 34
7.5. SAFETY MONITORING....34

8 RECORDING AND REPORTING OF ADVERSE EVENTS....35

8.1 DEFINITIONS 35
8.2 CAUSALITY ASSESSMENT....35
8.3 EXPECTEDNESS EVALUATION....36
8.4 RECORDING ADVERSE EVENTS 36
8.5 REPORTING SERIOUS ADVERSE EVENTS (SAE)....37
8.6 EXPEDITED REPORTING OF SUSAR....37
8.7 ANNUAL SAFETY REPORT....37
8.8 EXPECTED ADVERSE EVENTS....37

9. ADMINISTRATIVE ASPECTS....40

9.1 DATA HANDLING, RECORD KEEPING, SAMPLE STORAGE 40
9.2 SAMPLE STORAGE 40
9.3 RECORD RETENTION....40
9.4 PATIENT CONFIDENTIALITY....40
9.5 MONITORING & AUDIT....41
9.6 AMENDMENTS TO STUDY DOCUMENTS....41
9.7 DEFINITION OF END OF STUDY & END OF STUDY REPORT 41
9.8 INSURANCE & INDEMNITY....41
9.9. DISSEMINATION OF RESULTS....42

REFERENCES 43

APPENDIX 1: MSCV-HSVTK/CD34 VECTOR....51

APPENDIX 2: BIORELIANCE TESTSING OF MCB AND CLINICAL BATCH 53

APPENDIX 3: T CELL TRANSDUCTION DATA 56

APPENDIX 4: STANDARD TEMPLATE FOR HSCT....60

APPENDIX 5 MONITORING SCHEDULE....62

ABBREVIATIONS USED

ACV	Aciclovir
ALL	Acute lymphoblastic leukaemia
AML	Acute myeloid leukaemia
APC	Antigen presenting cell
BMT	Bone marrow transplantation
CFSE	Carboxy-fluorescein diacetate succinimidyl ester
CML	Chronic myeloid leukaemia
CMV	Cytomegalovirus
CTL	Cytotoxic T lymphocyte
DC	Dendritic cell
DLA	Dog leukocyte antigen
DLI	Donor leukocyte infusion
EBV	Epstein Barr Virus
FACS	Fluorescence activated cell sorting
FCS	Fetal calf serum
FISH	Fluorescent in situ hybridisation
FITC	Fluoroscein isothiocyanate
GCV	Ganciclovir
GM-T cell	Genetically modified T cell
GVHD	Graft versus host disease
GTAC	Gene therapy advisory committee
rIL-2	Recombinant human Interleukin-2
HD	Hodgkins disease
HLA	Human leukocyte antigen
HSCT	Haematopoietic stem cell transplantation
HSVTK	Herpes Simplex Virus thymidine kinase
scHSVTK	Splice corrected HSVTK
IVIG	Intravenous immunuglogulin
ΔLNGFR	Truncated low affinity nerve growth factor receptor
MESV	Murine embryonic stem cell virus
MHRA	The medicines and healthcare products regulatory agency
MLV	Murine leukaemia virus
MPSV	Myeloproliferative sarcoma virus
MM	Multiple myeloma
MOI	Multiplicity of infection
NHL	Non Hodgkins leukaemia
PHA	Phytohaemagglutinin
RN	Retronectin
tCD34	Truncated CD34

1. LAY SUMMARY

Bone marrow or blood stem cell transplantation is used to treat a wide range of life-threatening conditions. T lymphocytes carried in the graft have powerful beneficial effects and play a vital role in the eradication of leukaemia and in fighting infection, but can also damage healthy tissues and cause graft-versus-host disease (GVHD).

To safeguard against GVHD, we propose modifying T cells to encode a ‘switch’ so that they can be eliminated if problems arise.

Children receiving half-matched (haploidentical) transplants from a parent are most likely to benefit from this strategy. At present these patients receive blood stem cells from a parent, but the T cells are removed because the risk of serious GVHD is unacceptable. This means that they are much more likely to suffer from life threatening infections or experience a relapse of leukaemia. We want to use gene therapy to produce “safe” T cells which can be used to strengthen the transplant and prevent these serious complications.

2. OBJECTIVES OF THE STUDY

1. Transduction of donor T cells with a retroviral vector encoding a suicide gene/selection marker fusion gene

2. Administration of genetically modified (GM) donor T cells to facilitate immune reconstitution following haploidentical HSCT

3. Evaluation of T cell engraftment kinetics and stability

4. Elimination of GM-T cells using Ganciclovir in the event of GVHD

5. Longitudinal evaluation of clinical effect in terms of immune reconstitution, leukaemic relapse, and survival.

3. ROLE OF T CELLS IN HSCT

Allogeneic haematopoietic stem cell transplantation (HSCT) is used to treat a range of haematological malignancies as well as primary immunodeficiencies, and certain metabolic disorders(1). Approximately one third of candidates being considered for HSCT have a suitable HLA matched sibling donor. Although matched unrelated volunteer donors are now available for upto 75% of Northern Europeans, full matches are much more difficult to achieve for patients from other ethnic backgrounds. Haploidentical parental and sibling donors are available for most patients, but major immunological disparities between the donor and recipient may give rise to significant graft versus host disease (GVHD). This is a T cell mediated phenomenon and is a major cause of morbidity and mortality, limiting the wider application of HSCT.

3.1. GVHD following allogeneic HSCT

3.1.1 Definition

Acute GVHD usually arises within 2 months of allogeneic HSCT and is graded on the basis of skin, liver and gastrointestinal involvement (See 6.5 for grading system). There are usually characteristic histologically identifiable changes, including mononuclear infiltrates, architectural disruption and cell death (2). Despite pharmacological immunosuppression to protect against GVHD, moderate to severe GVHD has been reported in 25-60% of matched related transplants, and 45-70% of matched unrelated HSCT (3).

GVHD is termed as chronic 100 days after transplantation, and may present with a variety manifestations, involving virtually any organ system. Common complications include lichenoid changes of the skin and mucous membranes, autoimmune phenomena, haematological cytopaenia and cellular and humoral immunodeficiency (4).

3.1.2 Pathogenesis

The pathogenesis of GVHD is complex but the central role of donor T cells is well established (2). Pre-transplant conditioning procedures initiate inflammatory processes leading to cytokine release and up-regulation of adhesion molecules and MHC class II molecules in the recipient. This enhances the allo-activation of donor helper T cells, leading to the recruitment of cytotoxic T cells, natural killer cells, and mononuclear cells. Cytokines such as IL-1 and TNFα mediate pathogenesis by inducing cellular damage and further inflammation. Donor T cells may be directly activated by host derived antigen presenting cells (APCs) or donor derived APCs that cross-present host antigens. APCs provide crucial co-stimulatory signals leading to T cell cytokine production. Th_1 cytokines (Interferon-γ and IL-2) are important mediators of acute GVHD, whereas under certain circumstances Th_2 cytokines (such as IL-4) may suppress GVHD (5).

3.1.3 Risk factors for GVHD

Risk factors for GVHD include Human Leukocyte Antigen (HLA) disparity, increasing age, viral infections, reduced immunosuppression and the nature of the primary condition. HLA compatibility strongly influences both the likelihood of sustained engraftment and the risk of developing GVHD**.** As HLA-matched family donors are often not available, mismatched family donors and matched

unrelated volunteer donors are increasingly being considered, and present an increased likelihood of GVHD (6). It has been calculated that the risk of acute GVHD using a fully HLA-matched unrelated donor equates to a one antigen mismatched related donor, and a one antigen mismatched unrelated donor equates to a two antigen mismatched related donor (2). Female donors are occasionally sensitized to Y-antigen minor histocompatibility antigens and in some studies have been associated with a higher relative risk of subsequent GVHD. Older patients may present an increased risk of acute GVHD and this may reflect a reduced potential for effective thymic education of T cells and a greater likelihood of pathogen colonization. Donor and recipient seropositivity to Cytomegalavirus (CMV), Herpes Simplex Virus (HSV) or Epstein Barr Virus (EBV) has been linked to increased GVHD, though the mechanisms involved are not entirely clear (Ferrara *et al*, 1997). There may be an increased risk of GVHD following peripheral blood stem cell therapy (PBSCT) compared to BMT, although the issue remains controversial (7). Meta-analyses have suggested that the relative risk of acute GVHD is 1.13 and chronic GVHD is 1.5 for PBSCT compared to BMT, and this may reflect the increased number of T cells carried in mobilised peripheral blood harvests (8).

3.2 Management of GVHD

A number of immunosuppressive agents are used for prophylaxis against T cell mediated adverse effects, including Methotrexate, Cyclosporin, Tacrolimus and Mycophenylate Mofetil. Steroids are widely used both as prophylaxis and treatment of GVHD having potent anti-inflammatory and immunosuppressive effects. Depletion of T cells from the donor graft is the most effective way to reduce GVHD, but is also associated with a number of adverse effects, and is discussed in detail below. The likelihood of GVHD can be reduced if the intensity of the conditioning regimen is reduced, for example by using non-myeloblative transplants. Less toxic preparative regimens are associated with reduced tissue damage and cytokine release, and residual host T cells counter donor T cell mediated effects (9).

In general the treatment of GVHD is centred on the use of immunosuppressive agents, such as Prednisolone and Cyclosporin. Most mild cases of acute GVHD respond well to a course of Prednisolone, in association with adequate serum concentrations of Cyclosporin. High dose Prednisolone may be required for more severe cases, and occasionally treatment with Anti-Thymocyte Globulin (ATG) is used. The use of such agents to control GVHD is associated with infective complications, delayed reconstitution and prolonged periods of immunodeficiency (2). More recently, the use of the monoclonal antibodies Infliximab and Dacluzimab, directed against TNFα and CD25 respectively, have been used with some success in steroid refractory GvHD (10;11).

3.3 Depletion of donor T cells from allogeneic grafts

The most effective strategy to prevent GVHD is the depletion of donor T cells (12). This can be achieved through the *in vivo* use of monoclonal antibodies such as CAMPATH-1H. Alternatively, the graft can be largely depleted of T cells if HSC are selected using CD34 based magnetic bead selection systems (13). Patients transplanted with unmodified whole marrow typically receive around 10^7 T cells/kg. GVHD is unlikely if the inoculum has less than $5x10^4$ $CD3^+$ cells/kg (14) and is more likely to be encountered above $5x10^5$ $CD3^+$ cells/kg (15). Direct comparisons between studies

reporting varying rates of GVHD are difficult because of differences in methodology, immunosuppressive regimens, and the underlying conditions being treated. For example, in HLA identical sibling transplantation the incidence of grade II-IV acute GVHD has recently been reported at between 4 and 11% following TCD transplantation (16). The International Bone Marrow Registry (IBMTR) reported data from leukaemia patients undergoing related or unrelated or HLA mismatched transplants and found GVHD rates of around 35% for TCD, and 57% for T cell replete grafts (17). Others have reported that the incidence of GVHD in the HLA mismatched setting, using TCD grafts combined with post transplant immunosuppression of around 16% (18) and, in one series, there were no cases of significant GVHD (19). The incidence of hepatic and pulmonary dysfunction (independent of GVHD) following TCD transplantation is also reduced compared to T cell replete transplantation, and this may be a reflection of the deleterious effects of medications used for preventing GVHD (20).

Despite the advantages of TCD in preventing GVHD, organ dysfunction, and transplant related mortality, this does not necessarily translate into improved long term. Limitations associated with TCD include graft failure, delayed immune reconstitution and increased frequency of infective complications including EBV-associated lymphoproliferative disease and relapse of malignancy.

3.3.1 Graft failure following T cell depletion

Graft failure may be an immediate, immune mediated rejection phenomenon or may occur following partial engraftment within the first two weeks of transplantation. Delayed graft failure can arise after several months and may be associated with donor-recipient sex disparity, older age, viral infection and absence of immunosuppression (21). Graft failure using non-TCD allogeneic HSCT was historically less than 5% in leukaemia patients (22;23). One large retrospective analysis in 1991 estimated that in HLA-identical transplants in leukaemic patients there may be a nine fold increased risk of graft failure for TCD compared to T cell replete transplantation between HLA identical siblings (24). In another report, approximately 30% of patients transplanted with T cell depleted mismatched marrow from a related donor suffered graft failure (25). Graft failure most likely arises because residual host lymphocytes are able to eliminate populations of donor cells. Donor T cells would usually protect the graft by removing any host lymphoid cells that may have survived the conditioning procedure, and this effect is lost in T cell depleted grafts. Approaches aimed at preventing graft failure include the use of more intensive conditioning or pre-transplant immunosuppression, larger doses of donor stem cells and less rigorous T cell depletion (2).

3.3.2. Delayed immune reconstitution

Transplantation is followed by a period of immunodeficiency often complicated by life threatening infections. Recovery of lymphoid cells following allogeneic HSCT is usually sequential, with NK cells emerging within 3 weeks, followed by B cells (3-6months) and T cells (3-12months) (1). Regeneration of the T cell pool is known to occur via both thymic and non-thymic pathways. Studies in mouse models have indicated that in recipients without a thymus, the T cell pool can regenerate exclusively from peripheral expansion of memory phenotype donor T cells (26). The CD8 compartment usually expands preferentially and the CD4:CD8 ratio may be inverted for many months after transplantation. Recovery in TCD grafts is delayed in comparison to unmanipulated grafts (27), and the repertoire limited (28) and functionally impaired (29). The impaired reconstitution of cellular immunity in the post

transplant period results in an increased risk of viral infections and associated complications. For example, the relative risk of Epstein Barr Virus (EBV) associated lymphoproliferative disease (LPD) is estimated to be up to seven times greater in recipients of HLA identical T cell depleted grafts compared to unmodified transplants (30;31). The observation that infusions of donor lymphocytes can induce remission in some patients with EBV- LPD underlines the importance of a rapid recovery of T cell immunity (32). Similarly, virus specific T cells can be generated *ex-vivo* and used to prevent or treat infections such as EBV and CMV in the post transplant period (33;34).

3.3.3 Relapse of leukaemia

Patients who have undergone HSCT for malignancy may suffer relapse of the primary condition. There is a 5 fold increased risk of relapse after TCD transplantation for Chronic myeloid leukaemia (CML) compared to non-TCD transplantation (35); (36), although this is less pronounced in the unrelated donor setting (37). In one landmark study, 2,254 patients receiving HLA-identical sibling transplants for Acute myeloid leukaemic (AML), Acute Lymphoblastic Leukaemia (ALL) and CML were grouped on the basis of T cell depleted and non-depleted transplantation after comparable anti-leukaemia therapies (38). Patients developing acute and then chronic GVHD had the lowest rates of relapse, whereas those with T cell depleted grafts were most likely to relapse.

3.4 Donor lymphocyte infusions

The pre-emptive infusion of donor T cells in the early post transplant period to support anti-tumour effects has been hampered by a high incidence of GVHD but later donor lymphocyte infusions (DLI), following relapse, have been more successful. A number of malignancies are susceptible to GVL effects of donor lymphocytes. Most notably, DLI can achieve durable remission in patients with chronic myeloid leukaemia (CML) who relapse into chronic phase disease after allogeneic HSCT (39-42). Other susceptible haematological malignancies with similarly indolent kinetics have been successfully treated using DLI, including Chronic Lymphocytic Leukaemia (CLL) and low grade lymphomas (9). Acute myeloid leukaemia (AML) (43), Multiple Myeloma (44), Hodgkins Disease and intermediate grade lymphomas are less susceptible but do respond to DLI (45). Anti-tumour responses have also been demonstrated in patients with renal cell carcinoma (46) and some breast cancer patients (47) following allogeneic HSCT. DLI therapy has not generally been successful in more aggressive conditions such as high-grade lymphomas or ALL (48).

Following studies in canine models, DLI has also been advocated to convert stable mixed chimaerism following HSCT into full donor chimaerism (49). Recently, the use of DLI following non-myeloablative procedures was found to be of benefit for relapse of malignancy or persisting disease with acceptable levels of toxicity (50). The major factor limiting the wider application of DLI is the development of GVHD, in particular in the HLA mismatched setting. The use of DLI is associated with the development of GVHD in 55-60% of chronic phase CML patients. In general, most responding patients who achieve sustained remission develop some degree of GVHD. However, GVHD may become severe (grades III-IV) in around 13% and is estimated to cause fatal complications in approximately 5% of subjects receiving DLI (51;52). Furthermore, DLI may precipitate pancytopaenia in the host, and, although this is unusual, transient aplasia may develop in up to 5% of patients (53).

DLI is generally not considered in the haploidentical setting because the risks of intractable GVHD are unacceptable. **Strategies to control the adverse effects of donor lymphocytes, whilst allowing beneficial effects, would allow the use of donor T cells in the haploidentical setting**

4. T CELL SUICIDE GENE THERAPY

One approach, which has been recently deployed as a means of selectively targeting T cells involved in GVHD, relies on the genetic modification of donor T cells to encode a suicide gene mechanism. Suicide gene therapies rely on the conversion of a non-toxic prodrug into an active toxic form by a gene-encoded enzyme system. This was first shown to be feasible as an anti-cancer strategy in 1986 in experiments which demonstrated that the genetic transfer of Herpes simplex thymidine kinase (HSVTK) to tumour cells conferred selective chemosensitivity to Ganciclovir (GCV) (54). The system has been used to control GVHD following HSCT in clinical trials using donor T cells transduced with a retroviral vector to carry the HSVTK gene (55).

The ideal requirements for T cell suicide gene therapy and proposed mechanisms for delivering such criteria have been reviewed, and are summarised in the Table below (56)

**Requirement**	**Rationale**	**Mechanism**
Stable integration of suicide gene into host cell genome	Maintain expression of suicide gene in daughter cells following T cell division	Retroviral Vectors
High proportion of cells expressing the suicide gene	Ideally all the infused cells should carry the suicide gene to ensure elimination of clones causing GVHD	Inclusion of selectable markers for the enrichment of transduced cells
Stable expression over time of a functional suicide gene	Retain susceptibility to prodrug	T cell tropic constructs which are resistant to silencing; Elimination of splice variants
Absence of replication competent vectors	Reduce risk of insertional mutagenesis	Use of replication incompetent virus
Retention of a complete functional T cell repertoire	Preservation of T cell function	Minimise ex-vivo activation and manipulation

4.1. The HSVTK/GCV suicide gene system

The prodrugs GCV and Aciclovir (ACV) are anti-viral agents that are widely used for the treatment of Cytomegalovirus (CMV) and Herpes Simplex virus infections. They are poor substrates for mammalian nucleoside kinases but are efficiently phosphorylated to the monophosphate form by Herpes Virus derived thymidine kinases. Further phosphorylation by cellular kinases results in the production of toxic triphosphate derivatives that become incorporated into host cell DNA, leading to the death of actively dividing cells (57). Binding of ACV-triphosphate leads to termination of DNA elongation, whereas GCV-triphosphate causes base pair mismatches and DNA fragmentation. Activated forms of both drugs inhibit cellular DNA polymerases and block DNA synthesis. The HSVTK/GCV suicide gene system has been widely studied in a variety of tumour systems, including central nervous system gliomas, prostate tumours and neuroblastomas (58).

4.2. Retroviral vectors

Recombinant viral vectors that have been rendered replication defective can be modified to encode genes of interest and retroviral systems are of particular interest for gene delivery to haematopoietic cells as the transgene is stably integrated into the target cell genome. This is essential for suicide gene transfer to cell systems with a rapid turnover, because as daughter cells are produced they each encode a copy of the transgene.

The wild type retrovirus genome comprises a 7-11kb of linear, single stranded RNA that has a 5'cap and 3'poly A structure. Three genes, *gag* (group specific antigen), *pol* (RNA dependent DNA polymerase) and *env* (envelope), are flanked by short repeat (R) sequences and unique elements that include reverse transcription enhancers and promoter sequences (termed U5 and U3). The genome is encased in an icosahedral protein shell along with viral enzymes (protease, reverse transcriptase, and integrase) and a matrix protein. The viral envelope comprises a spherical lipid bilayer studded with glycoproteins derived from the infected virus-producing cell. The envelope glycoproteins mediate adhesion to cell surface receptors, leading to fusion with the cellular membrane. Following entry into the target cell, the viral genome is reverse transcribed from RNA to DNA using the reverse transcriptase enzyme carried by the virus. Long terminal repeat (LTR) regions are generated through the transposition and duplication of unique region sequences U3 and U5. As the cell divides the nuclear membrane disrupts and the proviral DNA integrates into the host cell genome.

Retroviral vectors reaching clinical evaluation to date have been derived from the Moloney Murine Leukaemia Virus (MoMLV). A number of *cis*-acting sequences must be retained within retroviral vector constructs including the 5' un-translated leader region containing a packaging signal and the outer regions of the LTRs, which are important for integration events. The primer binding site and repeat sequences, required for reverse transcription, are also retained but other regions of the viral genome can be excised and replaced by genes of interest of up to a maximum of 9kb in size. Promoter sequences in the U3 region of the 5'LTR can be used to drive transcription of the transgene, or alternatively an internal promoter can be included. Modifications of such regions and the primer binding site leader sequence have led to the generation of a variety of retroviral vectors with increased tropism and sustained transgene expression in T cells. The missing retroviral structural proteins are provided in specially constructed packaging cell lines that have been genetically modified to produce *gag, pol* and *env* components. The vector proposed for use in this study is was constructed by Fehse et al and incorporates a fusion protein combining HSVTK (splice site corrected) and truncated CD34 (59) (described in section 5.1) as a bi-functional pro-drug activating and cell selection system.

Truncated CD34 (tCD34), is an alternative splice variant of CD34 (a cell surface glycophosphoprotein expressed on HSC and small vessel endothelia) which has a truncated cytoplasmic tail of just 16 amino acids and lacks protein kinase C signaling domains (60). Fehse *et al*. (2000) have previously investigated the feasibility of using tCD34 to select retrovirally transduced T cells(61). They found that cells engineered to express tCD34 could be readily enriched using magnetic beads conjugated to an anti-CD34 antibody. The system has a major advantage in terms of both commercial and regulatory considerations. Approved reagents and equipment suitable for the separation of cells based on CD34 expression are well established and are regularly used in the clinical setting for the selection of $CD34^+$ HSC.

4.3. PG13 packaging cell line

The PG13 is derived from the murine fibroblast line NIH 3T3 and provides the Murine Leukaemia Virus (MLV) derived gag-pol components and the Gibbon Ape Leukaemia Virus (GALV) *env* cassette in a split packaging system (62). The GALV envelope binds the sodium-dependent phosphate symporter Pit-1 which is expressed at higher levels than Pit-2 (the amphotropic receptor) on haematopoietic cells. GALV pseudotyped viruses do not infect murine cells and thus are unlikely to lead to auto-infection of the packing cell line or multiple integration events which may otherwise lead to recombination events with replicative components of the viral genome. Although there is a finite risk of generating replication-competent retroviruses (RCR) from this cell line, this phenomenon has never been reported. Screening of cells and vector stocks during production and after CD34+ cell transduction will ensure that the clinical product is free from RCR and patients will be screened at regular intervals after infusion of transduced cells.

The PG13 line has been used to produce vector for clinical use for trials of SCID-X1, ADA deficiency and CGD in the UK. It has also been approved by regulatory authorities in the USA for vector production. The clinical grade retroviral supernatant used for this study has been prepared from a fully tested PG13 derived MCB, under GMP conditions, at Kings College London (KCL).

4.4 Procedures for the transduction of T cells

Primary T cell transduction with retroviral vectors requires pre-stimulation of the cells sufficient to cause cell division. The T cell mitogen PHA was used in early studies to induce primary T cell proliferation and enhance retroviral infection (63;64). More recently anti-CD3 antibody alone (65-69) or in combination with anti-CD28 antibody (70-74) has proven very effective. In addition a wide range of supplemental Interleukin (IL-2), with concentrations ranging between 25 and 1000u/ml, have been used. In our studies, primary T cells can be efficiently transduced following 48-72 hours activation with anti-CD3 (OKT3) and anti-CD28 antibodies, in combination 30iu/ml of IL-2 (75).

The *ex-vivo* activation, transduction and selection (in particular extended selective culture for antibiotic resistance) has been demonstrated to influence the repertoire and immunophenotype distribution of T cells (76). Some investigators have reported a profound reversal of the CD4/CD8 subset ratio after stimulation with anti-CD3 and IL-2 (77) and skewing of the T cell receptor Vβ repertoire. In contrast, activation with a combination of anti-CD3 and anti-CD28 monoclonal antibodies appears to preserve the existing T cell receptor Vβ repertoire (78).

GALV pseudotyped virus particles transduce T cells at high efficiency when used in combination with retronectin (79). This is the active CH-296 carboxy-terminal chymotryptic moiety of fibronectin and contains binding sites for integrins VLA-4 and VLA-5 (present on T cells) and a heparin-binding domain, which interacts with cell surface proteoglycans. A number of *in vitro* studies have documented enhanced retroviral transduction efficiency with the use of Retronectin. Clinical grade Retronectin is available and in clinical studies Retronectin (RN) coated gas permeable bags have been used to transduce HSC for the corrective gene therapy of X-linked severe combined immunodeficiency (SCID-X1) and ADA deficiency.

4.5 Animal studies

Animal models have been used to support the principle of T cell suicide gene therapy *in vivo* and to evaluate protocols for the timing of T cell infusions and GCV administration following HSCT. A transgenic mouse model in which a truncated, but functional, HSVTK gene is expressed in mature T cells has been invaluable in 'proof of principle' studies (80). Infusions of transgenic T cells into irradiated MHC mismatched allogeneic mice following bone marrow grafting induced GVHD. A 7 day course of Ganciclovir treatment at the time of the T cell infusion was sufficient to prevent GVHD without impairing haematological reconstitution. When chronic GVHD did develop in a small number of animals it was responsive to further treatment with Ganciclovir. The GCV treated mice maintained a pool of functional donor T cells that were tolerant to recipient alloantigens but responsive to third party alloantigens.

The ability of donor T cells to facilitate engraftment and cause GVHD can be dissociated by manipulating survival of allo-activated transgenic T cells through temporal variation of Ganciclovir administration (81). Optimal outcomes were achieved by treatment with GCV on days 3-16 post-infusion and reduced levels of GVHD were complimented by increased numbers of T cells in subsequent weeks. It appears that most alloreactive donor T cells divide before non-alloreactive cells and it is possible, at least in murine systems, to selectively eliminate the most harmful T cell fractions through the timely administration of GCV (82). Similar observations have been reported by Kornblau et al using retrovirally transduced murine T cells encoding HSVTK in an allograft model of GVHD, with optimal survival in groups receiving GCV between days 7-13 post transplant (83). Experiments in a murine transplant model of acute lymphoblastic leukaemia have also shown that donor HSVTK modified T cells can eradicate leukaemic cells without causing GVHD if the timing of GCV administration and dose of T cell incoculum are optimal (84).

A canine model of allogeneic bone marrow transplantation has been used to investigate the ability of retrovirally transduced and *ex-vivo* expanded cytotoxic T cells (CTLs) to enhance engraftment in the haploidentical transplantation setting (85). Donor-derived, recipient specific, CTLs were generated by establishing a one-way mixed lymphocyte cultures with cells from Dog Leukocyte Antigen (DLA) haploidentical littermates. Murine retroviral vectors encoding eGFP or rat nerve growth factor receptor and Neo^R gene, were pseudotyped with the GALV envelope and used to transduce the CTL populations. Twelve DLA-haploidentical recipients received transplants of unmodified donor marrow and CTLs (transduced or non transduced) after preparative total body irradiation (TBI) and in the absence of any immunosuppression. The infusion of CTLs prevented graft rejection and there was no statistical difference in engraftment between dogs receiving genetically modified and non-modified CTLs, and similar levels of GVHD were observed in both groups. Similarly, HSVTK transduced canine T cells have shown to retain allo-reactivity and were susceptible to GCV mediated elimination (86).

4.6 Human Clinical Trials of T cell suicide gene therapy

The first trial of HSVTK transduced T cells in humans was in patients with HIV infection. Genetically modified autologous T cells specific for the HIV–*gag* protein were administered to HIV infected patients without any acute toxic complications (87). A retroviral vector based on the MLV and

encoding a HSVTK/Hygromycin phosphotransferase fusion protein was used to transduce the cells but it was later demonstrated that host cytotoxic T cell responses against the fusion protein resulted in immune mediated clearance in 5/6 patients.

In 1997, Bonini *et al*. reported the results from a pilot study involving 8 patients who had relapsed after allogeneic HSCT for malignancy or had developed post transplant Epstein Barr virus driven lymphoproliferative disease (EBV-LPD) (88). A retroviral vector (SFCMM-2) encoding an HSVTK/Neo^R fusion protein and the cell surface marker, ΔLNGFR (truncated low affinity nerve growth factor receptor), was used to transduce donor T cells. The group co-cultured donor peripheral blood mononuclear cells (PBMC), activated with the mitogen phytohaemagglutinin (PHA), with an irradiated amphotropic packaging cell line. Transduced cells were selected by immunomagnetic bead selection using monoclonal antibodies to ΔLNGFR. Between 10^5/kg and $4x10^7$/kg GM-T cells were given as part of a dose escalating protocol (the threshold for causing GVHD is usually above $5x10^5$/kg (89). The percentage of circulating GM-T cells ranged between 0.0001% and 13.4%, and cells were detectable over 20 months later. There were three complete responses and two partial responses following the treatment. Three cases of acute GVHD were reported and 2/3 responded to a course of GCV, with elimination of GM-T cells from the circulation. The third patient had extended multisystem chronic GVHD, which did not respond to GCV therapy.
Ciceri et al (90) subsequently extended this experience in a phase I-II, multicentre, non-randomised trial of haploidentical stem-cell transplantation, and infused donor lymphocytes expressing the herpes-simplex thymidine kinase suicide gene after transplantation. The primary study endpoint was immune reconstitution defined as circulating $CD3^+$ count of 100 cells per μL on more for two consecutive observations. 50 patients (median age 51 years, range 17-66) received haploidentical stem-cell transplants for high-risk leukaemia. 28 patients received HSVTK-cells starting 28 days after transplantation; 22 patients obtained immune reconstitution at median 75 days (range 34-127) from transplantation and 23 days (13-42) from infusion. Ten patients developed acute GVHD (grade I-IV) and one developed chronic GVHD, which wascontrolled by induction of the suicide gene. Overall survival at 3 years was 49% (95% CI 25-73) for 19 patients who were in remission from primary leukaemia at the time of stem-cell transplantation. No acute or chronic adverse events were related to the gene-transfer procedure. Infusion of HSVTK cells was considered effective in accelerating immune reconstitution, while controlling GVHD and protecting patients from late mortality after haploidentical stem-cell transplantation.

A second group that has published results from pilot studies of T cell suicide gene therapy used amphotropic virus supernatant-based gene transfer protocols (91). PBMC were activated with anti-CD3 antibody and high concentrations of IL-2 (1000iu/ml). Transduced cells were selected on the basis of Neo^R gene co-expression and the transduction procedure required 12 days of *ex-vivo* manipulation. Using the G1TkSvNa vector, the transduction efficiency before G418 selection, as measured by quantitative PCR for Neo^R was around 8%. After selection 87% of cells were sensitive to GCV. Twelve patients with haematological malignancies underwent HLA-identical sibling BMT and received infusions of between $2x10^5$ and $2x10^6$ GM-T cells/kg at the time of transplantation. Of three patients who developed acute GVHD, two responded to GCV therapy and one required additional treatment with steroids. One case of chronic GVHD also responded to GCV therapy. Resolution of

GVHD was associated with a rapid reduction in the number of circulating GM-T cells. Although there were no acute toxic side effects, three patients subsequently developed EBV-LPD. These subjects were considered to be at high risk for developing EBV-LPD, on the basis of their age, the intensive conditioning received, and the use of immunosuppressive agents. Overall, 4/12 patients were alive at the time of publication (29-34 months post BMT), and circulating GM-T cells were detectable for over 800 days in some patients.

There have been limited reports from other groups undertaking clinical trials of T cell suicide gene therapy. Burt et al. reported results from nine patients with relapsed haematological malignancies after HSCT (92). One patient with cutaneous T cell lymphoma developed GVHD, which responded to GCV therapy. The overall incidence of GVHD following T cell infusions was much lower than would have been expected with non-transduced cell infusions and thus strategy of GCV mediated T cell elimination was not fully tested.

4.7 Immune response to HSVTK

Immune responses against a fusion construct of HSVTK and hygromycin have been reported (Riddell 1996) and responses against the Neo^R gene product was suspected in five of the patients treated by Bonini et al.(93). This component was deleted in later vector constructs. Berger et al demonstrated cell mediated responses against various HSVTK epitopes leading to clearance of gene modified T cells in vivo (94). The majority of patients in this study will be profoundly immunosuppressed and are unlikely to mount strong responses in the early post transplant period.

4.8 Ganciclovir Resistance

Partial resistance to GCV elimination was observed in initial clinical trials (95;96). PCR and southern blot analysis has detected the presence of an alternative splice variant of HSVTK in T cells recovered from a number of patients. Cells carrying this truncated, non functional, form of HSVTK accounted for around 10% of the T cell population and were present as an increasing proportion following the GCV elimination of the cells encoding full-length HSVTK (97). It was reported that cryptic splice donor and acceptor sites within the HSVTK open reading frame were responsible for the generation of a truncated, non-functional, splice variant form of the HSVTK in GCV resistant cells. The 227bp deletion was detected in cell lines (9-15%) and primary cells (upto 10%) transduced with the SFCMM3 vector (coding HSVTK and ΔLNGFR, and packaged using the GP+envAM12 amphotropic line) or the G1TkScNa vector (encoding HSVTK and the neomycin resistance selection gene and packaged using the PA317 line). It is postulated that alternative splicing events within the packaging cell line led to the production of retroviral particles encoding the truncated HSVTK form.

The construct developed for our study has been modified to eliminate the cryptic splice sites (and is termed splice corrected HSVTK or scHSVTK).

4.9 Functional potential of transduced T cells

Lower than expected rates of GVHD and infective complications observed in initial trials have raised questions about the functional capacity of infused lymphocytes, and in particular their ability to

mediate GVL and protect against viral infections. It has been proposed that the process of pre-activation, exposure to retrovirus and selection of transduced T cells may influence subsequent immune function. The nature of the mitogenic activation appears to be crucial. The use of anti-CD3 antibodies and IL-2, followed by G418 selection has been shown *in vitro* to impair anti-EBV potential (98;99) and this has been borne out in one clinical study where a number of subjects developed EBV related LPD (100). The addition of costimulation via anti-CD28 prevented culture related loss of EBV reactive T cells, probably by inducing anti-apoptotic effects and prevention of activation induced cell death caused by anti-CD3/IL-2 stimulation. The EBV reactive pool of T cells can also be better preserved if the period of *ex-vivo* culture is kept to a minimum by using early immuno-magnetic based selection for a co-expressed cell surface marker rather than using longer chemotherapy-selection based selection protocols.

The impact of the initial activation stimulus is highlighted by CFSE tracking experiments demonstrating how effectively cells are activated with anti-CD3/anti-CD28/30iu IL-2 (101). T cells complete the first cycle of cell division between 48 and 72 hours, and by the end of the procedure, six days later, cells have usually undergone over seven divisions. Phenotype analysis at this stage shows that over 90% of cells express CD3 and a small number (2-3%) of natural killer cells of the CD16+CD56$^+$ phenotype are seen. The CD4:CD8 ratio is usually reduced by the end of the transduction procedure and as expected, the cells are highly activated with most expressing the CD25 (IL-2 receptor-α) surface molecule. A majority of cells are of the memory phenotype (CD45RO+CD27+).

Analysis of the T cell receptor complementarity determining region-3 (CDR3) size analysis has been used to investigate the T cell repertoire before and after retroviral transduction. Reverse transcriptase-polymerase chain reaction (RT-PCR) of RNA extracted from T cells allows determination of the hypervariable Vβ chain CDR3 transcript length. Clonal expansion or deletion of T cells can be detected through distortions of the usually Gaussian-like distribution generated for each of the 24 Vβ subfamilies. Skewing of the CD4 and CD8 Vβ repertoire has also been noted in protocols that used a combination of anti-CD3 antibody and IL-2 to preactivate T cells for retroviral mediated gene transfer (102). The changes were not related to the process of retroviral transduction itself, but rather to the activation procedure and length of *ex-vivo* culture. Using a combination of anti-CD3/CD28/30iu IL-2, activated cells retain a Gaussian distribution and alterations in the T cell Vβ repertoire are not readily seen (103).

Studies in a rat model have suggested that the potential of activated and cultured T cells to mediate GVHD is reduced, in particular if they are manipulated for more than 6 days. Weijtens *et al.* investigated the potential of retrovirally transduced, ΔLNGFR-selected, rat T cells to mediate GVHD in MHC mismatched transplantation (104). T cells activated with Con A, transduced and magnetically selected within 5 days retained the ability to cause GVHD but cells cultured for longer than six days had a reduced capacity to induce GVHD. Similarly, Kornblau *et al.* showed that murine T cells activated using ConA and retrovirally transduced were prone to exhaustion if cultured for an extended period (105). We propose the early selection of transduced cells using magnetic beads conjugated to antibodies against the cells surface marker protein tCD34 (which is fused to the HSVTK suicide gene) and resting of the cells in culture before re-infusion. **This allows rapid selection and enrichment of transduced cells and minimise the loss of functionality that has been associated with extended culture periods.**

SUMMARY OF PREVIOUS CLINICAL TRIALS AND LIMITATIONS ENCOUNTERED

1. Clinical trials have established the feasibility of manipulating donor T cells *ex vivo* for the purposes of retroviral transduction with a suicide gene
2. Administration of GM-T cells can treat relapsed leukaemia after HSCT
3. The elimination of GM-T cells encoding HSVTK using GCV has been demonstrated.
4. A small fraction of GM-T cells encoded a non-active splice variant of HSVTK that was resistant to GCV
5. The pre-activation of T cells for retroviral transduction with PHA or OKT3 impaired their subsequent function *in vivo*
6. In some patients, immune responses were generated against the GM-T cells

RATIONALE FOR THE NEW CLINICAL TRIAL

1. We plan to use an improved retroviral vector for stable, long term gene expression in T cells of a scHSVTK/tCD34 fusion protein
2. Use of a splice corrected HSVTK gene reduce the risk of GCV resistant variants
3. Selection based on the truncated CD34 surface marker allows the use of existing clinical grade CliniMACs cell selection systems and is highly effective
4. Acitivation of T cells with a combination of beads conjugated to anti-CD3 and CD28 antibodies is a more physiological stimulus and allows greater preservation of T cell repertoire and functionality
5. All procedures to be performed in closed bag systems and using media free from non-human serum.
6. We propose the elective use of programmed GM-T cell infusions after haploidentical transplantation in children to boost immune reconstitution. These procedures are better tolerated in children compared to adults.

5. PROCEDURE FOR THE PREPARATION OF GM-T CELLS

5.1 Construction of retroviral vector

The retroviral vector backbone combines promotor elements from MPSV and a 5' untranslated region of MESV termed leader 71 (106). The vector encodes a suicide/selection fusion protein comprising splice corrected HSVTK and truncated human CD34. It has been shown to highly efficacious for the transduction, selection and GCV mediated elimination of human T cells. A schematic map of the plasmid and the sequence of the construct are shown in Appendix 1. A similar vector has recently been approved for use in a study of WT1 specific T cell receptor transfer in man (EudraCT: 2006-004950-25; IMP: pMP71-WT1 TCR alpha-2A-beta-Cys1).

5.2. Production of GALV pseudotyped virus

The production of clinical grade virus supernatant to GMP standards was carried out at Kings College London (KCL). A master cell bank comprising the PG13 packaging cell to pseudotype GALV enveloped virus at a titres >10^5/ml has been established. The virus supernatant and the master cell bank undergoes comprehensive testing for RCR and microbiological contamination by Biorelance.

5.3. T cell Transduction

PBMC will be prepared by centrifugation of 300-500ml of whole blood or leucapheresis product obtained from the donor. The cells will be resuspended at 10^6/ml in X-Vivo 10 supplemented with 10% human AB serum and 100 iu/ml of recombinant human IL-2 (all of these reagents are available as clinical grade materials). T cell activation will be carried out in clinical grade approved gas-permeable closed bag systems (available from Miltenyi Biotec) using clinical grade (Dynal) microbeads conjugated with anti-CD3 and anti-CD28 antibodies for 48 hours. The cells will then be counted and resuspended at 10^6/ml. Virus loading is performed by centrifugation of 50ml of viral supernatant in the retronectin coated bag. 50 ml samples will be transferred to bags that have been previously coated with Retronectin and pre-loaded with virus. The activated T cells will be exposed to viral supernatant for 48 hours and then washed and resuspended in X-Vivo 10 (see Appendix 3 for sample protocol).

5.4. Efficiency of CD34 selection

Transduced T cells will be selected using the CliniMACs systems according to the manufacturers' guidelines. This system is fully approved for human and is routinely deployed at our institute. Cells will be coated with Miltenyi microbeads, conjugated to anti-CD34 antibody for 60 minutes in a closed bag system. After washing, transduced cells will be magnetically selected and resuspended in X-VIVO 10. Viable cells will be counted by trypan blue exclusion. An aliquot of cells will be stained with PE-conjugated anti-CD34 antibody to assess the efficiency of the selection. If <90% of the selected population express CD34, the sample will be subjected to a second round of selection.

5.5. Molecular analysis of transduced cells

Transduced cells will be retained for analysis of sites of retroviral intergration and serial samples stored as indiacated on the monitoring schedule in Appendix 5. Integration site analysis will be performed on batches of cells using LAM-PCR and high throughput sequencing.

5.6. Function of suicide gene

Suicide gene function in transduced T cells plated in triplicates using a 96 well plate will be assessed by exposure to a gradient of Ganciclovir concentrations (0-100μM) over a 3 day period. The assay is well established in our laboratory and uses spectrophotometric analysis to quantify cell viability based on mitochondrial function. Survival of T cells transduced with HSVTK should be <20% at concentrations of GCV 10μM using the MTT assay, and no viable cells should be detected on trypan blue staining.

5.7 IMP Accountability and Traceability

HSVTK Transduced T cells will be manufactured in the GMP gene therapy loboratory, Great Ormond Street Hospital for Children.

Accountability of the IMP at site will be responsibility of the PI or delegate. Accountability logs will be maintained at site to document shipment, receipt, storage, administration and destruction of all IMPs. The site will ensure that there is a system in place to ensure traceability of the IMP to the patient.

6. HSVTK TRANSDUCED DONOR T LYMPHOCYTE INFUSIONS FOLLOWING HAPLO-IDENTICAL TRANSPLANTAION

6.1. Rationale

Almost all children and many adults have at least one haploidentical relative who may serve as a donor T cell depletion, the likelihood of infective complications and relapse of malignancy remain major obstacles. In Europe, longitudinal studies have calculated that the 3 year survival following HLA mismatched HSCT for primary immunodeficiencies is approximately 54% for SCID and 42% for non-SCID disorders (107). Survival at 18 months following haploidentical HSCT for childhood haematological disorders is currently estimated to be around 44% (range 27-71%) (108). Outcomes following haploidentical procedures in adult subjects are even poorer, often with less than 30% event free survival (Aversa 1998). Donor lymphocyte infusions in the haploidentical setting has been associated with GVHD and significant morbidity. **Thus genetic modification of haploidentical donor T cells with a suicide gene mechanism would allow them to be safely given after transplantation to boost immune reconstitution and reduce the risk of serious infections, disease relapse or graft failure.**

6.2. Statement of Aims

To demonstrate that HSVTK GM-T cells safely improve cellular immune reconstitution in children after haplo-identical HSCT

6.3 Patient population

We aim to treat **5-10** children in this study.

All patients with primary immunodeficiencies (including severe combined immunodeficiency, reticular dysgenesis, combined immunodeficiency, Wiskott-Aldrich, CD40L deficiency, Chronic granulomatous disease, Leukocyte Adhesion deficiency, Reticluar dysgenesis), Bone marrow failure syndromes (including Fanconi anaemia, Dyskeratosis congenita, Diamond Blackfan, Schwachman, Kostmann, Aplastic aneamia) metabolic diseases (Mucopolysaccharidoses, Osteopetrosis, Adrenoleukodystrophy, Metachromic leukodystrophy, Gauchers disease) and haematological malignancies (Acute and chronic myloid leukaemias, Acute lymphoblastic leukaemias and mylodysplastic syndromes) undergoing haplo identical transplant will be eligible.

6.3.1 Inclusion criteria (as for HSCT)

1. Patients with primary immunodeficiencies or haematological malignancies at GOSH (children of both sexes aged zero to 16 years) undergoing haplo identical transplant
2. Both patient (or parent/guardian) and donor must give informed consent in writing.
3. The donor must be willing, able and available for donation of cells by collection of whole blood or leucapheresis.
4. The patient should be free of serious intercurrent illness.

6.3.2 Exclusion criteria

1. Donor unfit or unavailable
2. Donor positive for Hepatitis B or C, or HTLV-1, or HIV
3. Patient receiving Ganciclovir, Aciclovir, Cidofovir a result of active CMV, adenovirus, varicella zoster or herpes simplex infection infection
4. GVHD ≥ grade II before infusion of GM- T cells (see 6.6)
5. Serious intercurrent illness

6.4. Conditioning regimes

Conditioning will be tailored to each individual patient based on the underlying condition. The majority of patients will receive a combination of the following agents.

Primary Immunodeficiencies/Metabolic diseases

Busulphan 14mg/kg (3.5mg/kg x 4)

Treosulphan 36-42mg/m^2

Melphalan 150mg/m^2

Fludarabine 150mg/m^2 (30mg/m^2 x 5)

Cyclophosphamide 200mg/kg (50mg/kg x 4)

ATG 20mg/kg (5mg/kg x 4)

Haematological diseases/leukaemia

Busulphan 14mg/kg (3.5mg/kg x 4)

Treosulphan 36-42mg/m^2

Fludarabine 125mg/m^2 (25mg/m^2 day -13 to -9)

Melphalan 150mg/m^2

TBI 1440cGy (180 cGy x 2# day -8 to -5)

Cyclophosphamide 120mg/kg (60mg/kg day -3 to -2)

ATG 20mg/kg (5mg/kg day -5 to -1)

6.5 Harvest of donor T cells

Harvest of donor lymphocytes will performed by the transplant team and will follow standard operating procedures for such collections

6.6.1 Time of GM-T cell infusion

Providing that there is no significant GVHD (grade II or greater) infusions will start at D+1 (D 0 -7 allowed) post haploidentical transplantation. The second dose will be given at least four to six weeks later after the first dose in the absence of GVHD (grade II or greater). If serotherapy has been used, it may be up to six week before the anti T cell effects have lapsed and thus infusion of the first dose will be delayed until day 40-47.

The minimum time interval between patients treated with gene modified cells will be four weeks

6.6.2 DOSE SCHEDULE AND ADMINISTRATION OF GM-T CELLS

GM-T cells will be given at a 1 month interval, providing that there is no significant GVHD

- *DOSE 1* *<$5x10^4$ cells/kg*
- *DOSE 2* *<$5x10^5$ cells/kg*

Baseline observations will be recorded, including blood pressure before infusion of GM-T cells and then at 15 minute intervals until administration is completed.

The cells will be transduced fresh and then cryopreserved for doses 1 and 2. The cryopreserved GM-T cells stored in bags will be sequentially thawed on the ward in a water bath. The cells will be infused over 5-10 minutes through a large vein peripherally or centrally through a Hickman line. In the event of any adverse reactions, chlorpheniramine and hydrocortisone will be given, as well as oxygen and salbutamol in the event of respiratory distress.

The second dose will be given one month after the first if the CD4 count remains <200 cells/ul (the level below which opportunistic infections are likely). Cells will only be given in the absence of GVHD (grade 2 or greater). The criteria for dose escalation is derived from other studies in the haploidentical setting using gene modified or allo-depleted cells in children.

Observations will continue at regular intervals for a minimum of 2 hours following infusion of the IMP. This is routine practice after blood and cell products.

Dosing will be limited to two infusions of gene modified T cells. Further doses will not be given if the CD4 count remains below 200 after the second infusion.

6.7 Treatment of subsequent CMV infection

Whole blood PCR will be performed as part of a routine program of monitoring for CMV, In the event of rising CMV viral loads on two consecutive occasions treatment will be initiated with Foscarnet. If the viral load continues to rise, Ganciclovir therapy will be initiated. This is likely to lead to the elimination of GM-T cells. Once CMV infection has been controlled, further infusions of GM-T cells may be indicated.

6.8 GvHD

GVHD will be scored according to the following criteria (Seattle)

STAGE	SKIN RASH	BILIRUBIN	DIARRHOEA
I	<25%	25-40 mmol/L	0.5-1.0L/day
II	25-50%	40-74	1-1.5
III	Generalised	75-200	>1.5
IV	Erythroderma, vesicles, exfoliation	>200	As above with pain or ileus

Individual patients will not receive the second dose if GVHD of grade II or greater has developed

The trial will be stopped in the event of GVHD that is not controlled with GCV therapy on the following basis.

No of patients on trial	4	5	6	7	8	9	10
No of patients presenting with GVHD after GCV	1	1	2	2	2	3	3

In previous clinical studies 1 of 3 patients treated with GCV had resistant GVHD, and with the improvements we have incorporated we anticipate much lower rates of refractory GVHD.

6.8.1 GVHD prophylaxis

Cyclosporin A (CsA) 3mg/kg starting day –1. Cyclosporin should have been discontinued at the time of infusion of GM-T cells. If not then the CyA dose will be tapered over 4 weeks (as long as GvHD does not develop) to improve T cell activity

6.8.2 Treatment of GVHD

In the event of grade II or III aGvHD patients will be given intravenous ganciclovir 5mg/kg b.d. If the condition progresses within 48 hrs or fails to improve within 7 days then 2mg/kg/day of methyl prednisolone will be given. In the event of grade IV GVHD a combination of Ganciclovir and methyl-prednisolone will be given. Further immunosuppression including additional prednisolone, CyA, Sirolimus, MMF, Tacrolimus, Methotrexate may be added at the discretion of the attending physician.

6.9 Clinical care

Great Ormond Street hospital for children has 9 in patient beds dedicated to children undergoing HSCT. In addition, there are over 30 beds for patients with primary immunodeficiencies, haematological disorders and malignancy. Day to day clinical management will be provided by the BMT medical and nursing teams

6.10 Laboratory follow-up

6.10.1 Routine monitoring

This will be performed as indicated in **Appendix 5**. This will include:

i. Measurement of full blood count, electrolyte profile, liver function tests, C-reactive protein.
ii. Surveillance for CMV,EBV and Adenovirus using quantitative PCR
iii. Analysis of donor chimaerism using PCR detection of polymorphic micro-satellite markers in sex matched transplants and FISH detection of X or Y chromosomes in sex mismatched procedures
iv. Quantification of tumour cell burden (Minimal residual disease, MRD) using PCR or cytogenetic analsysis
v. Analysis of T cell receptor repertoire and diversity
vi. Analysis of anti–viral T cell immunity

6.10.2 Tracking T cells

Blood will be collected (10-20ml, maximum 1ml/kg) before and after each infusion of GM-T cells, and at monthly intervals thereafter

Flow cytometry will be used to identify $CD3^{+}CD34^{+}$ GM-T cells, and such cells will be further characterised for memory or effector phenotypes based on the expression of CD45RO, CD27 and CCR7.

Quantitative PCR based analysis will be used to detect GM-T cells if the frequency of cells detected by flow cytometry is <1%.

If patients undergo tissue biopsy as part of their clinical management, samples will be analysed by immuno-staining for the presence of $CD3^{+}CD34^{+}$ T cell infiltration.

6.11.Outcome measures

The endpoint of the study will be 12 months after administration of the final dose of gene modified cells.

Laboratory studies outlined in section 6 will be used to establish the following specific measures of outcome:

1. Percentage of donor cell engraftment (chimaerism studies)
2. Kinetics of T cell reconstitution (CD4+ cells >300/mm^3, CD3+ cells>500/mm^3), mitogen responses and antigen specific immune recovery. Kinetics of B cell recovery as indicated by endogenous immunoglobulin production (IgM)
3. Virus specific T cell immunity as measured by tetramer specific T cells and ELISPOT analysis
4. T cell repertoire (by spectratype and Vβ repertoire analysis)
5. Incidence of GvHD following GM-T cell infusion
6. Efficacy of Ganciclovir in treating GvHD
7. Frequency, severity and duration of infective complications
8. Disease activity (MRD)
9. Survival at 12 months post transplant

A positive result for this study would be demonstrated if patients achieve stable full donor chimerism, reconstitute T cell immunity (CD4>300) and clear viral reactivations and do not suffer relapse of malignacy. In addition, successful exploitation of the suicide gene mechanism will be determined by successful control of GVHD with the prodrug Ganciclovir.

6.12. Withdrawal/Replecement of individual subjects

A patient can withdraw or be withdrawn from protocol treatment in the study at any time from enrolment until the transduced T cells have been administered. Subjects who wish to discontinue from the study at any time are free to do so. However the reasons for discontinuation should be documented by the investigator if possible. Patients who withdraw from the study after administration of the transduced T cells will be encouraged to have follow-up investigations so that the consequences of the administration can be documented and analysed. Any patient withdrawn prior to administration of transduced T cells will be replaced in the study.

6.13. Off study criteria

A patient will be considered off study under the following circumstances:

1. The patient is withdrawn from the study prior to administration of transduced T cells
2. The patients/parent/guardian withdraws consent for study procedures and data collection.
3. The patient is lost to follow-up.

6.14 Follow-up of withdrawn subjects

Patients withdrawn from the study prior to administration of transduced T cells and patients for whom consent has been withdrawn for the study, will resume normal clinical care. Efficacy and safety assessments will not be carried out from the point of withdrawal and data will not be recorded in the Case report forms (CRFs).

Premature termination of the study

Termination of the study will occur under the following circumstances:

a) Expiry or exhaustion of vector stocks
b) Overall stopping of trial after consultation with Gene Therapy Advisory Committee (GTAC) and the Medicines and Healthcare products Regulatory Agency (MHRA) (i.e. due to SUSARS occurring on trial).

6.15. Statistical considerations

A maximum of 10 patients will be enrolled into the trial and all evaluable patients will be included in any analyses. Formal statistical analyses is not possible in this small cohort of diverse patients, therefore data analyses will be descriptive in nature.

7. SAFETY

7.1 Risk to the donor

7.1.1 Leukaphaesis/Blood donation

Collection of lymphocyte by leukapharesis requires the insertion of two large bore cannulas. A local anaesthetic will be applied to minimise discomfort. There is a small risk of localized bruising at the site of cannulation. The volume of sample collected will be around 250ml on two consecutive days. Collection of blood (300-500ml) may cause localized bruising.

7.2 Risks to the recipient

7.2.1 Risks of insertional mutagenesis

In humans, there have been over 40 clinical trials involving retroviral gene delivery involving hundreds of subjects without reports of adverse insertional events (109). Following retroviral gene therapy for SCID-X1 leukaemic changes in five infants who had received autologous HSC transduced to express the IL-2 common-γ chain. In most cases, retroviral insertion close to the LIM domain only-2 (LMO2) gene, a known T cell oncogene, is thought to have induced uncontrollable proliferation of HSC derived lymphoid progenitor cells (110-113). It is at present unclear whether in these subjects the adverse impact of the insertional localisation was compounded by signals mediated by the common-γ chain transgene(114-116). Furthermore, in this group of profoundly immunodeficient patients, abnormal T cell clones may have escaped immune surveillance within an environment supportive to rapid expansion.

A murine model has recently been employed to investigate insertional mutagenesis following the retroviral transduction of HSC. The combinational effect of insertional activation of proto-oncogenes and other signalling genes was confirmed and an association with high copy number established (117). In both human and animal studies, retroviral insertional mutagenesis has only been observed in studies involving the modification of HSCs. Importantly, there have no reported cases of insertional mutagenesis in patients followed-up over an extended period following treatment with retrovirally transduced mature lymphocytes. Children treated for Adenosine deaminase deficiency with infusions of gene modified T cells have been shown to have detectable numbers of circulating T cells over a decade after undergoing gene therapy (118). Long-term persistence of GM-T cells has also been reported in previous trials of T cell suicide gene therapy (119) with no reports of leukaemic transformation of the manipulated cell populations. It should be emphasised that the use of vectors encoding a suicide gene confers an inherent safety mechanism and provides important protection against the uncontrolled proliferation of cells with leukaemic potential.

7.2.2 Risks of using a truncated CD34 selection marker

There is a possibility that tCD34 has potential to alter signalling in T cells or direct tissue homing. However, in murine models there is no evidence that tCD34 expression of T cells alters function or influences homing patterns.

7.2.3 Germline transmission of vector sequences

T cells are manipulated and transduced *ex vivo*, and extensively washed prior to re-infusion. No viral particles should be present in the T cell inoculum given to patients. The risk of gene transfer to other tissues, including gonads, is considered to be negligible and has not been seen in other clinical retroviral vector based studies.

7.2.4 Quality control of harvest and transduction process

Manipulation of cells *ex vivo* is potentially associated with contamination. However, all manipulations will be undertaken in a dedicated clinical gene therapy laboratory which has been built at the Institute of Child Health and Great Ormond Street Hospital NHS Trust and which has been used for our gene therapy studies on X-SCID and ADA deficiency. All manipulations will follow clinically acceptable guidelines. To minimise contamination, procedures are conducted within closed culture bag systems and cells are selected using the clinically approved Miltenyi Biotech Clinimacs system. Cells will be tested for microbial contamination prior to re-infusion.

7.2.5 Handling of transduced cells

The reports from clinical gene transfer studies and our own experience shows that the infusion of cultured and gene altered autologous blood progenitors is not associated with any significant reactions. There have been some cases of moderate allergic reactions including temporary drops in blood pressure and trouble breathing that may have been a result of trace amounts of substances (particularly the bovine serum components) present in the culture medium. In this study, the medium used to culture T cells is free of animal serum. (X-VIVO10/ 10% human AB serum). Extensive washing of cells before infusion will further reduce the risk of reactions to components in the culture medium. However, infusion of any type of blood cell product can be associated with reactions resulting from clumping of these cells or other immediate reactions related to sticking of these cells to blood vessels in the lungs. Reactions are treated by stopping the infusion and providing oxygen, antihistamines, steroids and medications or fluids to increase blood pressure. One of the important safety features of this gene therapy protocol is that T cells will be cultured in a sealed bag culture system. Gram stains and routine cultures will be performed on cell samples the day before infusion and on the day of infusion. If microorganisms are detected, the procedure will be immediately terminated.

7.2.6 Lack of GM-T cell efficacy

Poor clinical responses following the administration of GM-T cells may indicate sub-optimal efficacy of the GM-T cells. This may be suspected under the following circumstances:-

i. Increase in tumour burden despite GM-T cell infusions under the dose escalation program
ii. Reduction in the percentage of donor cell engraftment on two consecutive occasions despite the dose escalation program
iii. An increase in viral load on three consecutive occasions for CMV, EBV or Adenovirus as detected on PCR based surveillance following the infusion of GM-T cells and despite the administration of antiviral agents (other than Ganciclovir).

Infusion of a non-modified DLI will be offered to patients in whom the efficacy of GM-T cells is considered to be sub-optimal and who have not suffered from GVHD≥grade II.

7.2.7 Failure of HSVTK/GCV system

In the event of GVHD ≥ grade II, GCV will be administered and actively dividing T cells should be eliminated with an improvement in clinical condition. Non-improvement in the severity of GVHD may represent a failure of the HSVTK/GCV system. A sample of GM-T cells will be collected from the patient and tested in vitro against a

gradient of Ganciclovir concentrations. The patient will be treated with additional immunosuppressants at the discretion of the attending physician to control GVHD.

7.2.8 Unexpected events or outcomes

If GM-T cells are suspected of causing complications or toxicity that can not be attributed to any other cause, GCV therapy will be initiated. All such events will be immediately reported as detailed in chapter 8.

7.3. Public Health Considerations

T cells are transduced *ex vivo* in a closed culture system. The vector does not contain replication competent viruses, and will not be shed from transduced cells. The potential for transmission of vector sequences to other persons is therefore extremely small.

7.4. Good Clinical practice & and ethical issues

Good Clinical Practice

The study will be conducted in accordance with the UK legislation on clinical trials, International Conference on Harmonisation (ICH) for Good Clinical Practice (GCP) E6(R1) and the appropriate regulatory requirement(s). Essential clinical documents will be maintained to demonstrate the validity of the study and the integrity of the data collected. A Trial Master File (TMF) will be established at the beginning of the study, maintained for the duration of the study and retained according to the appropriate regulations.

Ethical Considerations

The study will be conducted in accordance with ethical principles founded in the World Medical Association Declaration of Helsinki, Ethical Principles for Medical Research Involving Human Subjects (found at http://www.wma.net/e/). The independent ethics committee GTAC will review all appropriate study documentation in order to safeguard the rights, safety and well-being of the patients. The protocol, Investigator's Brochure(s), informed consent, written information given to the patients, safety updates, annual progress reports, and any revisions to these documents will be provided to GTAC by the investigator.

7.5. Safety monitoring

We have not set up an Independent Monitoring Committee, however the safety aspects of the trial are monitored closely by the Chief Investigator and various trial staff, ans are ducsussed at monthly Trial Meetings. Urgent Matters (i,e, SAEs are discussed immediately in an appropriate forum and later the entire team is updated at these regular meetings.

8 RECORDING AND REPORTING OF ADVERSE EVENTS

8.1 Definitions

Adverse Event (AE)

This is defined as any untoward medical occurrence in the patient administered an IMP that does not necessarily have a causal relationship with this treatment.

Adverse Reaction (AR)

This is defined as any untoward and unintended response in a subject to an IMP, which is related to any dose administered to that subject.

Serious Adverse Event (SAE)

A serious adverse event is defined as any untoward medical occurrence in the patient administered an IMP, which does not necessarily have a causal relationship with this treatment, and that at any dose:

- Results in death
- Is life threatening
- Results in persistent or significant or disability/incapacity
- Requires in-patient hospitalisation or prolongs existing hospitalisation
- Results in a congenital abnormality or birth defect
- Medically significant (i.e. any event which the investigator considers significant but which is not covered by the above.)

Suspected Serious Adverse Reaction (SSAR)

This is defined as an adverse reaction that is classed in nature as serious and which is consistent with the information about the IMP in question as set out in the Investigators brochure.

Suspected Unexpected Serious Adverse Reaction (SUSAR)

The definition of a SUSAR is a serious adverse drug reaction, the nature or severity of which is not consistent with the applicable product information (Investigators brochure) or which adds significant information on the specificity or severity of an expected event.

8.2 Causality assessment

When recording and reporting adverse events the relationship of that event to the medicinal product should be determined according to the following classification.

Not Related:	the adverse event is not reasonably related to the medicinal product / intervention - or another cause can itself explain the occurrence of the event
Unlikely Related:	the adverse event is doubtfully related to the medicinal product / intervention but can’t be fully ruled out.

Possibly Related:	the adverse event is reasonably related to the medicinal product / intervention, but the event could have been due to another, equally likely cause
Probably Related:	the adverse event is reasonably related to the medicinal product / intervention, and the event is more likely explained by the medicinal product / intervention than by any other cause
Definitely Related:	the adverse event is clearly related to the medicinal product / intervention and there is no other cause to explain the event or a re-challenge (if feasible) is positive

Events assessed as possibly, probably or definitely related to the cell therapy are considered adverse reactions (ARs).

8.3 Expectedness evaluation

The expectedness of an SAE/SSAR will be determined with reference to the Investigators Brochure. The event will be considered unexpected if it is not listed in the IB or if it adds significant information on the specificity or severity of an expected event. Sometimes, unexpected adverse drug reactions and unexpected serious adverse events become 'expected' during the trial, in which case the protocol will be amended and such events would not need reporting. The Sponsor and Chief Investigator and Data Monitoring Committee, will determine whether any events become 'expected' during the course of the trial and apply for MHRA and Ethics Committee approval for a substantial amendment.

8.4 Recording adverse events

At each scheduled visit, adverse events that might have occurred since the previous visit or assessment will be elicited from the patient/parent/guardian.

The investigators will maintain a record of all adverse events/occurrences in patients participating in the clinical trial. This record will be noted in the patient's medical notes.

Adverse events that have a causal relationship to the IMP (ARs) and SAEs will be recorded on the AE reporting section of the CRF. Occasions may arise where an independent medical expert opinion is required. For example when assess 'relatededness' of the IMP to and event which may influence the outcome of the clinical trial. In such an event an independent clinician will the review the data and make decision. Two paediatricians are named below who have agreed to act in such a capacity:

Dr Robert Wynn, Consultant Paediatric Haematologist, Director Bone Marrow Transplant Unit
Royal Manchester Children's Hospital
Oxford Road
Manchester
M13 9WL
0161 701 8417

Professor Irene Roberts
Professor of Paediatric Haematology

Department of Haematology, Division of Investigative Sciences Hammersmith and St Mary's Faculty of Medicine Imperial College of Science, Technology and Medicine Hammersmith Hospital Du Cane Road London W12 0NN
Tel: 0208 383 2163/4017

8.5 Reporting serious adverse events (SAE)

Events defined as serious will be reported in as much detail as possible to the sponsor (GOSH R&D office) **within 24-48 hours** of the investigator observing or learning of the event, unless a longer timeframe has been specified below (see front cover for sponsor contact details).

This notification will be recorded on the sponsors SAE report form and faxed within the reporting timelines.

The Investigator may be asked by the sponsor to provide follow-up information which should be reported on Sponsor follow-up form.

Serious adverse events 'related' to the protocol treatment will be followed up until resolved or considered stable (see section 11.2 Causality assessment). Follow-up will take place during hospital visits or by telephone should the patient's period of treatment/follow-up at GOSH terminate.

8.6 Expedited reporting of SUSAR

SAE reported to the sponsor that is evaluated by the Investigator as related to the IMP and unexpected will be reported to the regulatory authorities (MHRA) and the ethics committee (GTAC) by the sponsor within the expedited reporting timelines:

Fatal or life threatening SUSARS:
not later than **7 days** after the sponsor has information that the case fulfills the criteria for a fatal or life threatening SUSAR, and any follow up information within a further 8 days.

All other SUSARs:
not later than **15 days** after the sponsor for pharmacovigilance has information that the case fulfills the criteria for a SUSAR.

8.7 Annual safety report

Within 60 days of the anniversary of the clinical trial authorisation (CTA) date an annual safety report will be sent to MHRA with copy to GTAC.

8.8 Expected adverse events

The following SAE's are considered related to haploidentical transplantation

1. Death from complications of the disease process
2. Hospitalisation from complications of the disease
3. Prolonged hospitalisation from complications of the disease

The following adverse events are <u>expected</u> after haploidentical stem cell transplantation

1. Cytopaenias (either autoimmune, drug-induced or due to hypoplastic graft) with resultant need for transfusion, risk of bleeding and infection
2. Infections (bacterial, fungal and viral)
3. Idiopathic pneumonitis
4. Veno-occlusive disease of the liver
5. Thrombotic thrombocytopaenic purpura
6. Seizures related to hypertension/ electrolyte imbalance/drugs
7. Haemorrhagic cystitis
8. Exacerbation of pre-existing enteropathy/pneumatosis intestinalis
9. Toxicities related to drugs given independent of the study including derangement of electrolytes, renal and liver function, hypertension
10. Rejection/secondary graft failure
11. Acute and chronic graft-versus-host disease occurring prior to or more than 8 weeks after infusion of transduced CTL
12. Mixed chimerism
13. Relapse of malignancy

The following AEs are considered expected reactions that could occur following administration of gene modified T cells:

1. Lymphoproliferation due to insertional mutagenesis (SSAE)
2. Chills/fevers/rigors at time of infusion
3. Graft versus host disease
4. Cytopenia

Time-lines for reporting expected SAE/Rs to the sponsor:

All expected SAE/SSARs (except those identified as critical to the safety evaluation of the study, see below) will be reported to the sponsor using the sponsor SAE form within **30 working days** of the investigator becoming aware of the event.

Medically important events considered critical to the safety evaluation of the study:

Abnormal transgene related lymphoproliferation (i.e. in the absence of viral reactivation) is considered critical to the safety evaluation of the study and will be reported **immediately** (within 24-48hrs) to the sponsor using the

sponsors SAE form. Such events will be reported to the regulatory authorities (MHRA) and the ethics committee (GTAC) by the sponsor within the expedited reporting timelines stated.

Patients undergoing HSCT are monitored long term, often for life. As a minimum there will be annual monitoring, and any emergence of lymphoproliferative disease will be reported to the sponsor, GTAC and MHRA in the same way as during the study.

9. ADMINISTRATIVE ASPECTS

9.1 Data Handling, Record Keeping, Sample Storage

CRF completion: Selected data will be recorded on study specific case report forms (CRF's). The CRFs will be completed in black ink only, by personnel authorised to enter or change data on the CRF.Corrections can be made by striking out errors, with a single stroke, and not using correction fluid or obscuring the original entry. The correct entry must be entered by the side and initialled and dated by the person authorised to make the correction.Clinical and Research laboratory results will be held electronically in the GOSH pathology system. The CRF will document when the patient samples have been taken and when tests have been carried out.

9.2 Sample storage

A record of retained body fluids / tissue samples will be completed every time a sample is stored. This includes the patient trial identification number initials, date sample was stored, and storage location as well as the date the sample was moved or destroyed.

Samples (Cells, DNA & RNA) will be stored pre cell therapy and at regular intervals post cell therapy, and kept indefinitely at the bone marrow laboratory GOSH (or alternative approved location). Should it be required, samples will be disposed of in the appropriate manner according to local GOSH procedures, this will be detailed in the parent/guardian information sheet and consent obtained.

9.3 Record retention

Essential documents will be retained for a **minimum** of 25 years after completion of the trial. These documents will be retained for longer if required by the applicable regulatory requirements or the sponsor.

Records related to traceability of the IMP at site along with the patient identification code list will be retained at site for atleast 30 years from the expiry date of the HSVTK transduced cells.

The sponsor (or CI if delegated) will retain a copy of the final clinical trial Protocol and Investigator Brochure along with the traceability records for the trial for atleast 30 years from the expiry date of the HSVTK transduced cells.

9.4 Patient confidentiality

In order to maintain patient privacy, all CRFs, IMP accountability records, study reports and communications will identify the patient by the assigned unique patient trial number, initials and date of birth.

Direct access to the patient's original medical records for verification of data gathered on the CRFs and to audit the data collection process will be permitted for trial-related monitoring & audits by the sponsor, REC review, and regulatory inspection(s).

9.5 Monitoring & Audit

The trial will be monitored and audited by the trial sponsor (GOSH) according to their Standard Operating Procedures.

9.6 Amendments to study documents

Amendments are changes made to the research after a favourable opinion by the independent ethics committee and regulatory authority has been given.

A ‘substantial amendment’ is defined as an amendment to the terms of the ethics committee or Clinical trial authorisation (CTA) application, or to the protocol or any other supporting documentation, that is likely to affect to a significant degree:

- the safety or physical or mental integrity of the subjects of the trial;
- the scientific value of the trial;
- the conduct or management of the trial; or
- the quality or safety of any intervention used in the trial.

All substantial amendments will be firstly reviewed by the sponsor and then notified to GTAC and the MHRA. Approval (where applicable) will be obtained prior to implementation of any changes.

Non-substantial amendments will not be notified to GTAC and the MHRA, but will be recorded.

9.7 Definition of end of study & end of study report

The sponsor will notify GTAC and the MHRA of the end of the study within a period of 90 days.

The end of the study is defined as the last patient’s last scheduled visit according to the protocol, which will be the 12 month followup of the last patient entered into the trial.

In case the study is ended prematurely, the sponsor will notify GTAC and the MHRA within 15 days, including the reasons for the premature termination.

Within one year after the end of the study, the investigator/sponsor will submit a final study report with the results of the study, including any publications/abstracts of the study, to GTAC and the MHRA.

9.8 Insurance & indemnity

Cover for negligent harm will be provided by the Great Ormond Street Hospital for Children NHS Trust through the Clinical Negligent Scheme for Trusts (CNST). No-fault compensation insurance cover for any non-negligent harm will be provided by University College London (UCL).

9.9. Dissemination of results

Results of this study will be disseminated by publication, oral presentation at scientific meetings, and by direct communication with regulatory agencies.

REFERENCES

(1) Barrett J, Treleaven J. The clinical practice of stem cell transplantation. 1998. ISIS MEDICAL MEDIA.

(2) Ferrara JL, Deeg HJ, Burakoff SJ. Graft-vs-Host Disease. 1997. New York, Dekker.

(3) Ho VT, Soiffer RJ. The history and future of T-cell depletion as graft-versus-host disease prophylaxis for allogeneic hematopoietic stem cell transplantation. Blood 2001; 98(12):3192-3204.

(4) Vogelsang GB. How I treat chronic graft-versus-host disease. Blood 2001; 97(5):1196-1201.

(5) Teshima T, Ferrara JL. Understanding the alloresponse: new approaches to graft-versus-host disease prevention. Semin Hematol 2002; 39(1):15-22.

(6) Erlich HA, Opelz G, Hansen J. HLA DNA typing and transplantation. Immunity 2001; 14(4):347-356.

(7) Korbling M, Anderlini P. Peripheral blood stem cell versus bone marrow allotransplantation: does the source of hematopoietic stem cells matter? Blood 2001; 98(10):2900-2908.

(8) Cutler C, Giri S, Jeyapalan S, Paniagua D, Viswanathan A, Antin JH. Acute and chronic graft-versus-host disease after allogeneic peripheral- blood stem-cell and bone marrow transplantation: a meta-analysis. J Clin Oncol 2001; 19(16):3685-3691.

(9) Storb RF, Champlin R, Riddell SR, Murata M, Bryant S, Warren EH. Non-myeloablative transplants for malignant disease. Hematology (Am Soc Hematol Educ Program) 2001;375-391.

(10) Couriel DR, Saliba R, Hicks K, Ippoliti C, De Lima MJ, Hosing C et al. Tumor necrosis factor alpha blockade for the treatment of steroid-refractory acute GVHD. Blood 2004.

(11) Srinivasan R, Chakrabarti S, Walsh T, Igarashi T, Takahashi Y, Kleiner D et al. Improved survival in steroid-refractory acute graft versus host disease after non-myeloablative allogeneic transplantation using a daclizumab-based strategy with comprehensive infection prophylaxis. Br J Haematol 2004; 124(6):777-786.

(12) Korngold B, Sprent J. Lethal graft-versus-host disease after bone marrow transplantation across minor histocompatibility barriers in mice. Prevention by removing mature T cells from marrow. J Exp Med 1978; 148(6):1687-1698.

(13) Ho VT, Soiffer RJ. The history and future of T-cell depletion as graft-versus-host disease prophylaxis for allogeneic hematopoietic stem cell transplantation. Blood 2001; 98(12):3192-3204.

(14) Aversa F, Tabilio A, Velardi A, Cunningham I, Terenzi A, Falzetti F et al. Treatment of high-risk acute leukemia with T-cell-depleted stem cells from related donors with one fully mismatched HLA haplotype. N Engl J Med 1998; 339(17):1186-1193.

(15) Ho VT, Soiffer RJ. The history and future of T-cell depletion as graft-versus-host disease prophylaxis for allogeneic hematopoietic stem cell transplantation. Blood 2001; 98(12):3192-3204.

(16) Hale G, Jacobs P, Wood L, Fibbe WE, Barge R, Novitzky N et al. CD52 antibodies for prevention of graft-versus-host disease and graft rejection following transplantation of allogeneic peripheral blood stem cells. Bone Marrow Transplant 2000; 26(1):69-76.

(17) Champlin RE, Passweg JR, Zhang MJ, Rowlings PA, Pelz CJ, Atkinson KA et al. T-cell depletion of bone marrow transplants for leukemia from donors other than HLA-identical siblings: advantage of T-cell antibodies with narrow specificities. Blood 2000; 95(12):3996-4003.

(18) Henslee-Downey PJ, Abhyankar SH, Parrish RS, Pati AR, Godder KT, Neglia WJ et al. Use of partially mismatched related donors extends access to allogeneic marrow transplant. Blood 1997; 89(10):3864-3872.

(19) Aversa F, Tabilio A, Velardi A, Cunningham I, Terenzi A, Falzetti F et al. Treatment of high-risk acute leukemia with T-cell-depleted stem cells from related donors with one fully mismatched HLA haplotype. N Engl J Med 1998; 339(17):1186-1193.

(20) Ho VT, Soiffer RJ. The history and future of T-cell depletion as graft-versus-host disease prophylaxis for allogeneic hematopoietic stem cell transplantation. Blood 2001; 98(12):3192-3204.

(21) Ho VT, Soiffer RJ. The history and future of T-cell depletion as graft-versus-host disease prophylaxis for allogeneic hematopoietic stem cell transplantation. Blood 2001; 98(12):3192-3204.

(22) Beatty PG, Clift RA, Mickelson EM, Nisperos BB, Flournoy N, Martin PJ et al. Marrow transplantation from related donors other than HLA-identical siblings. N Engl J Med 1985; 313(13):765-771.

(23) Powles RL, Morgenstern GR, Kay HE, McElwain TJ, Clink HM, Dady PJ et al. Mismatched family donors for bone-marrow transplantation as treatment for acute leukaemia. Lancet 1983; 1(8325):612-615.

(24) Marmont AM, Horowitz MM, Gale RP, Sobocinski K, Ash RC, van Bekkum DW et al. T-cell depletion of HLA-identical transplants in leukemia. Blood 1991; 78(8):2120-2130.

(25) Ash RC, Horowitz MM, Gale RP, van Bekkum DW, Casper JT, Gordon-Smith EC et al. Bone marrow transplantation from related donors other than HLA- identical siblings: effect of T cell depletion. Bone Marrow Transplant 1991; 7(6):443-452.

(26) Mackall CL, Granger L, Sheard MA, Cepeda R, Gress RE. T-cell regeneration after bone marrow transplantation: differential CD45 isoform expression on thymic-derived versus thymic-independent progeny. Blood 1993; 82(8):2585-2594.

(27) Keever CA, Small TN, Flomenberg N, Heller G, Pekle K, Black P et al. Immune reconstitution following bone marrow transplantation: comparison of recipients of T-cell depleted marrow with recipients of conventional marrow grafts. Blood 1989; 73(5):1340-1350.

(28) Roux E, Helg C, Dumont-Girard F, Chapuis B, Jeannet M, Roosnek E. Analysis of T-cell repopulation after allogeneic bone marrow transplantation: significant differences between recipients of T-cell depleted and unmanipulated grafts. Blood 1996; 87(9):3984-3992.

(29) Soiffer RJ, Bosserman L, Murray C, Cochran K, Daley J, Ritz J. Reconstitution of T-cell function after CD6-depleted allogeneic bone marrow transplantation. Blood 1990; 75(10):2076-2084.

(30) Zutter MM, Martin PJ, Sale GE, Shulman HM, Fisher L, Thomas ED et al. Epstein-Barr virus lymphoproliferation after bone marrow transplantation. Blood 1988; 72(2):520-529.

(31) Gerritsen EJ, Stam ED, Hermans J, van den BH, Haraldsson A, van Tol MJ et al. Risk factors for developing EBV-related B cell lymphoproliferative disorders (BLPD) after non-HLA-identical BMT in children. Bone Marrow Transplant 1996; 18(2):377-382.

(32) Papadopoulos EB, Ladanyi M, Emanuel D, Mackinnon S, Boulad F, Carabasi MH et al. Infusions of donor leukocytes to treat Epstein-Barr virus-associated lymphoproliferative disorders after allogeneic bone marrow transplantation. N Engl J Med 1994; 330(17):1185-1191.

(33) Rooney CM, Smith CA, Ng CY, Loftin SK, Sixbey JW, Gan Y et al. Infusion of cytotoxic T cells for the prevention and treatment of Epstein-Barr virus-induced lymphoma in allogeneic transplant recipients. Blood 1998; 92(5):1549-1555.

(34) Gustafsson A, Levitsky V, Zou JZ, Frisan T, Dalianis T, Ljungman P et al. Epstein-Barr virus (EBV) load in bone marrow transplant recipients at risk to develop posttransplant lymphoproliferative disease: prophylactic infusion of EBV-specific cytotoxic T cells. Blood 2000; 95(3):807-814.

(35) Goldman JM, Gale RP, Horowitz MM, Biggs JC, Champlin RE, Gluckman E et al. Bone marrow transplantation for chronic myelogenous leukemia in chronic phase. Increased risk for relapse associated with T-cell depletion. Ann Intern Med 1988; 108(6):806-814.

(36) Marmont AM, Horowitz MM, Gale RP, Sobocinski K, Ash RC, van Bekkum DW et al. T-cell depletion of HLA-identical transplants in leukemia. Blood 1991; 78(8):2120-2130.

(37) Hessner MJ, Endean DJ, Casper JT, Horowitz MM, Keever-Taylor CA, Roth M et al. Use of unrelated marrow grafts compensates for reduced graft-versus- leukemia reactivity after T-cell-depleted allogeneic marrow transplantation for chronic myelogenous leukemia. Blood 1995; 86(10):3987-3996.

(38) Horowitz MM, Gale RP, Sondel PM, Goldman JM, Kersey J, Kolb HJ et al. Graft-versus-leukemia reactions after bone marrow transplantation. Blood 1990; 75(3):555-562.

(39) Kolb HJ, Schattenberg A, Goldman JM, Hertenstein B, Jacobsen N, Arcese W et al. Graft-versus-leukemia effect of donor lymphocyte transfusions in marrow grafted patients. European Group for Blood and Marrow Transplantation Working Party Chronic Leukemia. Blood 1995; 86(5):2041-2050.

(40) Drobyski WR, Hessner MJ, Klein JP, Kabler-Babbitt C, Vesole DH, Margolis DA et al. T-cell depletion plus salvage immunotherapy with donor leukocyte infusions as a strategy to treat chronic-phase chronic myelogenous leukemia patients undergoing HLA-identical sibling marrow transplantation. Blood 1999; 94(2):434-441.

(41) Porter DL, Roth MS, McGarigle C, Ferrara JL, Antin JH. Induction of graft-versus-host disease as immunotherapy for relapsed chronic myeloid leukemia. N Engl J Med 1994; 330(2):100-106.

(42) Guglielmi C, Arcese W, Dazzi F, Brand R, Bunjes D, Verdonck LF et al. Donor lymphocyte infusion for relapsed chronic myelogenous leukemia: prognostic relevance of the initial cell dose. Blood 2002; 100(2):397-405.

(43) Porter DL, Collins RH, Jr., Hardy C, Kernan NA, Drobyski WR, Giralt S et al. Treatment of relapsed leukemia after unrelated donor marrow transplantation with unrelated donor leukocyte infusions. Blood 2000; 95(4):1214-1221.

(44) Lokhorst HM, Schattenberg A, Cornelissen JJ, Thomas LL, Verdonck LF. Donor leukocyte infusions are effective in relapsed multiple myeloma after allogeneic bone marrow transplantation. Blood 1997; 90(10):4206-4211.

(45) van Besien K, Sobocinski KA, Rowlings PA, Murphy SC, Armitage JO, Bishop MR et al. Allogeneic bone marrow transplantation for low-grade lymphoma. Blood 1998; 92(5):1832-1836.

(46) Childs R, Chernoff A, Contentin N, Bahceci E, Schrump D, Leitman S et al. Regression of metastatic renal-cell carcinoma after nonmyeloablative allogeneic peripheral-blood stem-cell transplantation. N Engl J Med 2000; 343(11):750-758.

(47) Ueno NT, Rondon G, Mirza NQ, Geisler DK, Anderlini P, Giralt SA et al. Allogeneic peripheral-blood progenitor-cell transplantation for poor- risk patients with metastatic breast cancer. J Clin Oncol 1998; 16(3):986-993.

(48) Collins RH, Goldstein S, Giralt S, Levine J, Porter D, Drobyski W et al. Donor leukocyte infusions in acute lymphocytic leukemia. Bone Marrow Transplant 2000; 26(5):511-516.

(49) Georges GE, Storb R, Thompson JD, Yu C, Gooley T, Bruno B et al. Adoptive immunotherapy in canine mixed chimeras after nonmyeloablative hematopoietic cell transplantation. Blood 2000; 95(10):3262-3269.

(50) Bethge WA, Hegenbart U, Stuart MJ, Storer BE, Maris MB, Flowers ME et al. Adoptive immunotherapy with donor lymphocyte infusions after allogeneic hematopoietic cell transplantation following nonmyeloablative conditioning. Blood 2004; 103(3):790-795.

(51) Collins RH, Shpilberg O, Drobyski WR, Porter DL, Giralt S, Champlin R et al. Donor leukocyte infusions in 140 patients with relapsed malignancy after allogeneic bone marrow transplantation. J Clin Oncol 1997; 15(2):433-444.

(52) Kolb HJ, Schattenberg A, Goldman JM, Hertenstein B, Jacobsen N, Arcese W et al. Graft-versus-leukemia effect of donor lymphocyte transfusions in marrow grafted patients. European Group for Blood and Marrow Transplantation Working Party Chronic Leukemia. Blood 1995; 86(5):2041-2050.

(53) Keil F, Haas OA, Fritsch G, Kalhs P, Lechner K, Mannhalter C et al. Donor leukocyte infusion for leukemic relapse after allogeneic marrow transplantation: lack of residual donor hematopoiesis predicts aplasia. Blood 1997; 89(9):3113-3117.

(54) Moolten FL. Tumor chemosensitivity conferred by inserted herpes thymidine kinase genes: paradigm for a prospective cancer control strategy. Cancer Res 1986; 46(10):5276-5281.

(55) Bonini C, Ferrari G, Verzeletti S, Servida P, Zappone E, Ruggieri L et al. HSV-TK gene transfer into donor lymphocytes for control of allogeneic graft-versus-leukemia. Science 1997; 276(5319):1719-1724.

(56) Qasim W, Gaspar HB, Thrasher AJ. T cell suicide gene therapy to aid haematopoietic stem cell transplantation. Curr Gene Ther 2005; 5(1):121-132.

(57) Greco O, Dachs GU. Gene directed enzyme/prodrug therapy of cancer: historical appraisal and future prospectives. J Cell Physiol 2001; 187(1):22-36.

(58) Fillat C, Carrio M, Cascante A, Sangro B. Suicide gene therapy mediated by the Herpes Simplex virus thymidine kinase gene/Ganciclovir system: fifteen years of application. Curr Gene Ther 2003; 3(1):13-26.

(59) Fehse B, Kustikova OS, Li Z, Wahlers A, Bohn W, Beyer WR et al. A novel 'sort-suicide' fusion gene vector for T cell manipulation. Gene Ther 2002; 9(23):1633-1638.

(60) Krause DS, Fackler MJ, Civin CI, May WS. CD34: structure, biology, and clinical utility. Blood 1996; 87(1):1-13.

(61) Fehse B, Richters A, Putimtseva-Scharf K, Klump H, Li Z, Ostertag W et al. CD34 splice variant: an attractive marker for selection of gene- modified cells. Mol Ther 2000; 1(5 Pt 1):448-456.

(62) Miller AD, Garcia JV, von Suhr N, Lynch CM, Wilson C, Eiden MV. Construction and properties of retrovirus packaging cells based on gibbon ape leukemia virus. J Virol 1991; 65(5):2220-2224.

(63) Mavilio F, Ferrari G, Rossini S, Nobili N, Bonini C, Casorati G et al. Peripheral blood lymphocytes as target cells of retroviral vector- mediated gene transfer. Blood 1994; 83(7):1988-1997.

(64) Bonini C, Ferrari G, Verzeletti S, Servida P, Zappone E, Ruggieri L et al. HSV-TK gene transfer into donor lymphocytes for control of allogeneic graft-versus-leukemia. Science 1997; 276(5319):1719-1724.

(65) Lam JS, Reeves ME, Cowherd R, Rosenberg SA, Hwu P. Improved gene transfer into human lymphocytes using retroviruses with the gibbon ape leukemia virus envelope. Hum Gene Ther 1996; 7(12):1415-1422.

(66) Movassagh M, Boyer O, Burland MC, Leclercq V, Klatzmann D, Lemoine FM. Retrovirus-mediated gene transfer into T cells: 95% transduction efficiency without further in vitro selection [In Process Citation]. Hum Gene Ther 2000; 11(8):1189-1200.

(67) Ayuk F, Li Z, K Kh, Lindemann C, Schade U, Eckert HG et al. Establishment of an optimised gene transfer protocol for human primary T lymphocytes according to clinical requirements. Gene Ther 1999; 6(10):1788-1792.

(68) Fehse B, Schade UM, Li Z, Uhde A, Koch S, Goller B et al. Highly-efficient gene transfer with retroviral vectors into human T lymphocytes on fibronectin. Br J Haematol 1998; 102(2):566-574.

(69) Tiberghien P, Ferrand C, Lioure B, Milpied N, Angonin R, Deconinck E et al. Administration of herpes simplex-thymidine kinase-expressing donor T cells with a T-cell-depleted allogeneic marrow graft. Blood 2001; 97(1):63-72.

(70) Rudoll T, Phillips K, Lee SW, Hull S, Gaspar O, Sucgang N et al. High-efficiency retroviral vector mediated gene transfer into human peripheral blood CD4+ T lymphocytes. Gene Ther 1996; 3(8):695-705.

(71) Yang S, Delgado R, King SR, Woffendin C, Barker CS, Yang ZY et al. Generation of retroviral vector for clinical studies using transient transfection. Hum Gene Ther 1999; 10(1):123-132.

(72) Ferrand C, Robinet E, Contassot E, Certoux JM, Lim A, Herve P et al. Retrovirus-mediated gene transfer in primary T lymphocytes: influence of the transduction/selection process and of ex vivo expansion on the T cell receptor beta chain hypervariable region repertoire. Hum Gene Ther 2000; 11(8):1151-1164.

(73) Dardalhon V, Jaleco S, Rebouissou C, Ferrand C, Skander N, Swainson L et al. Highly efficient gene transfer in naive human T cells with a murine leukemia virus-based vector. Blood 2000; 96(3):885-893.

(74) Ferrand C, Robinet E, Contassot E, Certoux JM, Lim A, Herve P et al. Retrovirus-mediated gene transfer in primary T lymphocytes: influence of the transduction/selection process and of ex vivo expansion on the T cell receptor beta chain hypervariable region repertoire. Hum Gene Ther 2000; 11(8):1151-1164.

(75) Qasim W, Thrasher AJ, Buddle J, Kinnon C, Black ME, Gaspar HB. T cell transduction and suicide with an enhanced mutant thymidine kinase. Gene Ther 2002; 9(12):824-827.

(76) Ferrand C, Robinet E, Contassot E, Certoux JM, Lim A, Herve P et al. Retrovirus-mediated gene transfer in primary T lymphocytes: influence of the transduction/selection process and of ex vivo expansion on the T cell receptor beta chain hypervariable region repertoire. Hum Gene Ther 2000; 11(8):1151-1164.

(77) Movassagh M, Boyer O, Burland MC, Leclercq V, Klatzmann D, Lemoine FM. Retrovirus-mediated gene transfer into T cells: 95% transduction efficiency without further in vitro selection [In Process Citation]. Hum Gene Ther 2000; 11(8):1189-1200.

(78) Qasim W, King D, Buddle J, Verfuerth S, Kinnon C, Thrasher AJ et al. The impact of retroviral suicide gene transduction procedures on T cells. Br J Haematol 2003; 123(4):712-719.

(79) Qasim W, Thrasher AJ, Buddle J, Kinnon C, Black ME, Gaspar HB. T cell transduction and suicide with an enhanced mutant thymidine kinase. Gene Ther 2002; 9(12):824-827.

(80) Cohen JL, Boyer O, Salomon B, Onclercq R, Charlotte F, Bruel S et al. Prevention of graft-versus-host disease in mice using a suicide gene expressed in T lymphocytes. Blood 1997; 89(12):4636-4645.

(81) Drobyski WR, Morse HC, III, Burns WH, Casper JT, Sandford G. Protection from lethal murine graft-versus-host disease without compromise of alloengraftment using transgenic donor T cells expressing a thymidine kinase suicide gene. Blood 2001; 97(8):2506-2513.

(82) Maury S, Salomon B, Klatzmann D, Cohen JL. Division rate and phenotypic differences discriminate alloreactive and nonalloreactive T cells transferred in lethally irradiated mice. Blood 2001; 98(10):3156-3158.

(83) Kornblau SM, Stiouf I, Snell V, Przepiorka D, Stephens LC, Champlin R et al. Preemptive control of graft-versus-host disease in a murine allogeneic transplant model using retrovirally transduced murine suicidal lymphocytes. Cancer Res 2001; 61(8):3355-3360.

(84) Drobyski WR, Gendelman M, Vodanovic-Jankovic S, Gorski J. Elimination of leukemia in the absence of lethal graft-versus-host disease after allogenic bone marrow transplantation. J Immunol 2003; 170(6):3046-3053.

(85) Georges GE, Storb R, Bruno B, Brodie SJ, Thompson JD, Taranova AG et al. Engraftment of DLA-haploidentical marrow with ex vivo expanded, retrovirally transduced cytotoxic T lymphocytes. Blood 2001; 98(12):3447-3455.

(86) Georges GE, Storb R, Brunvand MW, Kiem HP, Moore PF, Malik P et al. Canine T cells transduced with a herpes simplex virus thymidine kinase gene: a model to study effects on engraftment and control of graft-versus-host disease. Transplantation 1998; 66(4):540-544.

(87) Riddell SR, Elliott M, Lewinsohn DA, Gilbert MJ, Wilson L, Manley SA et al. T-cell mediated rejection of gene-modified HIV-specific cytotoxic T lymphocytes in HIV-infected patients. Nat Med 1996; 2(2):216-223.

(88) Bonini C, Ferrari G, Verzeletti S, Servida P, Zappone E, Ruggieri L et al. HSV-TK gene transfer into donor lymphocytes for control of allogeneic graft-versus-leukemia. Science 1997; 276(5319):1719-1724.

(89) Ho VT, Soiffer RJ. The history and future of T-cell depletion as graft-versus-host disease prophylaxis for allogeneic hematopoietic stem cell transplantation. Blood 2001; 98(12):3192-3204.

(90) Ciceri F, Bonini C, Stanghellini MT, Bondanza A, Traversari C, Salomoni M et al. Infusion of suicide-gene-engineered donor lymphocytes after family haploidentical haemopoietic stem-cell transplantation for leukaemia (the TK007 trial): a non-randomised phase I-II study. Lancet Oncol 2009.

(91) Tiberghien P, Ferrand C, Lioure B, Milpied N, Angonin R, Deconinck E et al. Administration of herpes simplex-thymidine kinase-expressing donor T cells with a T-cell-depleted allogeneic marrow graft. Blood 2001; 97(1):63-72.

(92) Burt RK, Drobyski WR, Seregina T, Traynor A, Oyama Y, Keever-Taylor C et al. Herpes simplex thymidine kinase gene-transduced donor lymphocyte infusions. Exp Hematol 2003; 31(10):903-910.

(93) Verzeletti S, Bonini C, Marktel S, Nobili N, Ciceri F, Traversari C et al. Herpes simplex virus thymidine kinase gene transfer for controlled graft-versus-host disease and graft-versus-leukemia: clinical follow-up and improved new vectors. Hum Gene Ther 1998; 9(15):2243-2251.

(94) Berger C, Flowers ME, Warren EH, Riddell SR. Analysis of transgene-specific immune responses that limit the in vivo persistence of adoptively transferred HSV-TK-modified donor T cells after allogeneic hematopoietic cell transplantation. Blood 2006; 107(6):2294-2302.

(95) Bonini C. Infusion of HSV-TK engineered donor lymphocytes after haplo-identical hemopoietic stem cell transplantation provides early immune reconstitution and selective treatment of GVHD. ASGT 2002. Molecular Therapy 5, 1353. 2002.

Ref Type: Generic

(96) Verzeletti S, Bonini C, Marktel S, Nobili N, Ciceri F, Traversari C et al. Herpes simplex virus thymidine kinase gene transfer for controlled graft-versus-host disease and graft-versus-leukemia: clinical follow-up and improved new vectors. Hum Gene Ther 1998; 9(15):2243-2251.

(97) Garin MI, Garrett E, Tiberghien P, Apperley JF, Chalmers D, Melo JV et al. Molecular mechanism for ganciclovir resistance in human T lymphocytes transduced with retroviral vectors carrying the herpes simplex virus thymidine kinase gene. Blood 2001; 97(1):122-129.

(98) Sauce D, Bodinier M, Garin M, Petracca B, Tonnelier N, Duperrier A et al. Retrovirus-mediated gene transfer in primary T lymphocytes impairs their anti-Epstein-Barr virus potential through both culture-dependent and selection process-dependent mechanisms. Blood 2002; 99(4):1165-1173.

(99) Sauce D, Rufer N, Mercier P, Bodinier M, Remy-Martin JP, Duperrier A et al. Retrovirus-mediated gene transfer in polyclonal T cells results in lower apoptosis and enhanced ex vivo cell expansion of CMV-reactive CD8 T cells as compared with EBV-reactive CD8 T cells. Blood 2003; 102(4):1241-1248.

(100) Tiberghien P, Ferrand C, Lioure B, Milpied N, Angonin R, Deconinck E et al. Administration of herpes simplex-thymidine kinase-expressing donor T cells with a T-cell-depleted allogeneic marrow graft. Blood 2001; 97(1):63-72.

(101) Qasim W, King D, Buddle J, Verfuerth S, Kinnon C, Thrasher AJ et al. The impact of retroviral suicide gene transduction procedures on T cells. Br J Haematol 2003; 123(4):712-719.

(102) Movassagh M, Boyer O, Burland MC, Leclercq V, Klatzmann D, Lemoine FM. Retrovirus-mediated gene transfer into T cells: 95% transduction efficiency without further in vitro selection [In Process Citation]. Hum Gene Ther 2000; 11(8):1189-1200.

(103) Qasim W, King D, Buddle J, Verfuerth S, Kinnon C, Thrasher AJ et al. The impact of retroviral suicide gene transduction procedures on T cells. Br J Haematol 2003; 123(4):712-719.

(104) Weijtens M, van Spronsen A, Hagenbeek A, Braakman E, Martens A. Reduced graft-versus-host disease-inducing capacity of T cells after activation, culturing, and magnetic cell sorting selection in an allogeneic bone marrow transplantation model in rats. Hum Gene Ther 2002; 13(2):187-198.

(105) Kornblau SM, Stiouf I, Snell V, Przepiorka D, Stephens LC, Champlin R et al. Preemptive control of graft-versus-host disease in a murine allogeneic transplant model using retrovirally transduced murine suicidal lymphocytes. Cancer Res 2001; 61(8):3355-3360.

(106) Fehse B, Kustikova OS, Li Z, Wahlers A, Bohn W, Beyer WR et al. A novel 'sort-suicide' fusion gene vector for T cell manipulation. Gene Ther 2002; 9(23):1633-1638.

(107) Antoine C, Muller S, Cant A, Cavazzana-Calvo M, Veys P, Vossen J et al. Long-term survival and transplantation of haemopoietic stem cells for immunodeficiencies: report of the European experience 1968-99. Lancet 2003; 361(9357):553-560.

(108) Veys P, Amrolia P, Rao K. The role of haploidentical stem cell transplantation in the management of children with haematological disorders. Br J Haematol 2003; 123(2):193-206.

(109) Kohn DB, Sadelain M, Dunbar C, Bodine D, Kiem HP, Candotti F et al. American Society of Gene Therapy (ASGT) ad hoc subcommittee on retroviral-mediated gene transfer to hematopoietic stem cells. Mol Ther 2003; 8(2):180-187.

(110) Hacein-Bey-Abina S, von Kalle C, Schmidt M, McCormack MP, Wulffraat N, Leboulch P et al. LMO2-associated clonal T cell proliferation in two patients after gene therapy for SCID-X1. Science 2003; 302(5644):415-419.

(111) Hacein-Bey-Abina S, von Kalle C, Schmidt M, Le Deist F, Wulffraat N, McIntyre E et al. A serious adverse event after successful gene therapy for X-linked severe combined immunodeficiency. N Engl J Med 2003; 348(3):255-256.

(112) Howe SJ, Mansour MR, Schwarzwaelder K, Bartholomae C, Hubank M, Kempski H et al. Insertional mutagenesis combined with acquired somatic mutations causes leukemogenesis following gene therapy of SCID-X1 patients. J Clin Invest 2008; 118(9):3143-3150.

(113) Hacein-Bey-Abina S, Garrigue A, Wang GP, Soulier J, Lim A, Morillon E et al. Insertional oncogenesis in 4 patients after retrovirus-mediated gene therapy of SCID-X1. J Clin Invest 2008; 118(9):3132-3142.

(114) Dave UP, Jenkins NA, Copeland NG. Gene therapy insertional mutagenesis insights. Science 2004; 303(5656):333.

(115) Woods NB, Bottero V, Schmidt M, von Kalle C, Verma IM. Gene therapy: therapeutic gene causing lymphoma. Nature 2006; 440(7088):1123.

(116) Thrasher AJ, Gaspar HB, Baum C, Modlich U, Schambach A, Candotti F et al. Gene therapy: X-SCID transgene leukaemogenicity. Nature 2006; 443(7109):E5-E6.

(117) Modlich U, Kustikova OS, Schmidt M, Rudolph C, Meyer J, Li Z et al. Leukemias following retroviral transfer of multidrug resistance 1 (MDR1) are driven by combinatorial insertional mutagenesis. Blood 2005.

(118) Muul LM, Tuschong LM, Soenen SL, Jagadeesh GJ, Ramsey WJ, Long Z et al. Persistence and expression of the adenosine deaminase gene for 12 years and immune reaction to gene transfer components: long-term results of the first clinical gene therapy trial. Blood 2003; 101(7):2563-2569.

(119) Tiberghien P, Ferrand C, Lioure B, Milpied N, Angonin R, Deconinck E et al. Administration of herpes simplex-thymidine kinase-expressing donor T cells with a T-cell-depleted allogeneic marrow graft. Blood 2001; 97(1):63-72.

APPENDIX 1: MSCV-HSVTK/CD34 VECTOR

The critical raw material is a retroviral vector called MSCV-HSVTKCD34. There is no recommended International Nonproprietary Name (INN). The MSCV retroviral vector incorporates long terminal repeat (LTR) sequences from MPSV and a leader sequence from MESV. The vector is pseudotyped with the GALV envelope.

MSCV: Murine stem cell virus
MESV: Murine embryonic stem cell virus
MPSV: Myeloproliferative sarcoma virus
tCD34: Truncated CD34
scHSVTK: Splice corrected Herpes simplex thymidine kinase
GALV: Gibbon ape leukaemia virus

The full sequence is provided.

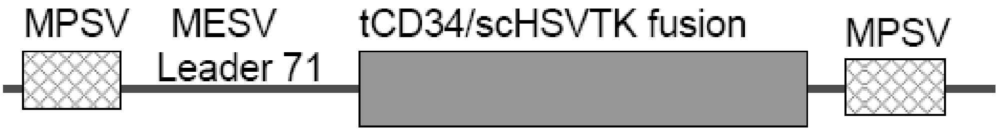


```
   1 AGCTTAACAC GAGCCATAGA TAGAATAAAA GATTTTATTT AGTCTCCAGA AAAAGGGGGG
  61 AATGAAAGAC CCCACCTGTA GGTTTGGCAA GCTAGCTTAA GTAACGCCAT TTTGCAAGGC
 121 ATGGAAAATA CATAACTGAG AATAGAGAAG TTCAGATCAA GGTTAGGAAC AGAGAGACAG
 181 CAGAATATGG GCCAAACAGG ATATCTGTGG TAAGCAGTTC CTGCCCCGGC TCAGGGCCAA
 241 GAACAGTTGG AACAGCAGAA TATGGGCCAA ACAGGATATC TGTGGTAAGC AGTTCCTGCC
 301 CCGGCTCAGG GCCAAGAACA GATGGTCCCC AGATGCGGTC CCGCCCTCAG CAGTTTCTAG
 361 AGAACCATCA GATGTTTCCA GGGTGCCCCA AGGACCTGAA ATGACCCTGT GCCTTATTTG
 421 AACTAACCAA TCAGTTCGCT TCTCGCTTCT GTTCGCGCGC TTCTGCTCCC CGAGCTCAAT
 481 AAAAGAGCCC ACAACCCCTC ACTCGGCGCG CCAGTCCTCC GATAGACTGC GTCGCCCGGG
 541 GTACCCGTGT TCTCAATAAA CCCTCTTGCA GTTTGCATCC GACTCGTGGT CTCGCTGTTC
 601 CTTGGGAGGG TCTCCTCTGA GTGATTGACT GCCCACCTCG GGGGTCTTTC ATTCTCGAGC
 661 AGCTTGGCGT AATCATGGTC ATAGCTGTTT CCTGTGTGAA ATTGTTATCC GCTCACAATT
 721 CCACACAACA TACGAGCCGG AAGCATAAAG TGTAAAGCCT GGGGTGCCTA ATGAGTGAGC
 781 TAACTCACAT TAATTGCGTT GCGCTCACTG CCCGCTTTCC AGTCGGGAAA CCTGTCGTGC
 841 CAGCTGCATT AATGAATCGG CCAACGCGCG GGGAGAGGCG GTTTGCGTAT TGGGCGCTCT
 901 TCCGCTTCCT CGCTCACTGA CTCGCTGCGC TCGGTCGTTC GGCTGCGGCG AGCGGTATCA
 961 GCTCACTCAA AGGCGGTAAT ACGGTTATCC ACAGAATCAG GGGATAACGC AGGAAAGAAC
1021 ATGTGAGCAA AAGGCCAGCA AAAGGCCAGG AACCGTAAAA AGGCCGCGTT GCTGGCGTTT
1081 TTCCATAGGC TCCGCCCCCC TGACGAGCAT CACAAAAATC GACGCTCAAG TCAGAGGTGG
1141 CGAAACCCGA CAGGACTATA AAGATACCAG GCGTTTCCCC CTGGAAGCTC CCTCGTGCGC
1201 TCTCCTGTTC CGACCCTGCC GCTTACCGGA TACCTGTCCG CCTTTCTCCC TTCGGGAAGC
1261 GTGGCGCTTT CTCAATGCTC ACGCTGTAGG TATCTCAGTT CGGTGTAGGT CGTTCGCTCC
1321 AAGCTGGGCT GTGTGCACGA ACCCCCCGTT CAGCCCGACC GCTGCGCCTT ATCCGGTAAC
1381 TATCGTCTTG AGTCCAACCC GGTAAGACAC GACTTATCGC CACTGGCAGC AGCCACTGGT
1441 AACAGGATTA GCAGAGCGAG GTATGTAGGC GGTGCTACAG AGTTCTTGAA GTGGTGGCCT
1501 AACTACGGCT ACACTAGAAG GACAGTATTT GGTATCTGCG CTCTGCTGAA GCCAGTTACC
1561 TTCGGAAAAA GAGTTGGTAG CTCTTGATCC GGCAAACAAA CCACCGCTGG TAGCGGTGGT
1621 TTTTTTGTTT GCAAGCAGCA GATTACGCGC AGAAAAAAAG GATCTCAAGA AGATCCTTTG
1681 ATCTTTTCTA CGGGGTCTGA CGCTCAGTGG AACGAAAACT CACGTTAAGG GATTTTGGTC
1741 ATGAGATTAT CAAAAAGGAT CTTCACCTAG ATCCTTTTAA ATTAAAAATG AAGTTTTAAA
1801 TCAATCTAAA GTATATATGA GTAAACTTGG TCTGACAGTT ACCAATGCTT AATCAGTGAG
1861 GCACCTATCT CAGCGATCTG TCTATTTCGT TCATCCATAG TTGCCTGACT CCCCGTCGTG
1921 TAGATAACTA CGATACGGGA GGGCTTACCA TCTGGCCCCA GTGCTGCAAT GATACCGCGA
1981 GACCCACGCT CACCGGCTCC AGATTTATCA GCAATAAACC AGCCAGCCGG AAGGGCCGAG
2041 CGCAGAAGTG GTCCTGCAAC TTTATCCGCC TCCATCCAGT CTATTAATTG TTGCCGGGAA
2101 GCTAGAGTAA GTAGTTCGCC AGTTAATAGT TTGCGCAACG TTGTTGCCAT TGCTGCTGGC
2161 ATCGTGGTGT CACGCTCGTC GTTTGGTATG GCTTCATTCA GCTCCGGTTC CCAACGATCA
2221 AGGCGAGTTA CATGATCCCC CATGTTGTGC AAAAAAGCGG TTAGCTCCTT CGGTCCTCCG
2281 ATCGTTGTCA GAAGTAAGTT GGCCGCAGTG TTATCACTCA TGGTTATGGC AGCACTGCAT
2341 AATTCTCTTA CTGTCATGCC ATCCGTAAGA TGCTTTTCTG TGACTGGTGA GTACTCAACC
2401 AAGTCATTCT GAGAATAGTG TATGCGGCGA CCGAGTTGCT CTTGCCCGGC GTCAATACGG
```

```
2461 GATAATACCG CGCCACATAG CAGAACTTTA AAAGTGCTCA TCATTGGAAA ACGTTCTTCG
2521 GGGCGAAAAC TCTCAAGGAT CTTACCGCTG TTGAGATCCA GTTCGATGTA ACCCACTCGT
2581 GCACCCAACT GATCTTCAGC ATCTTTTACT TTCACCAGCG TTTCTGGGTG AGCAAAAACA
2641 GGAAGGCAAA ATGCCGCAAA AAAGGGAATA AGGGCGACAC GGAAATGTTG AATACTCATA
2701 CTCTTCCTTT TTCAATATTA TTGAAGCATT TATCAGGGTT ATTGTCTCAT GAGCGGATAC
2761 ATATTTGAAT GTATTTAGAA AAATAAACAA ATAGGGGTTC CGCGCACATT TCCCCGAAAA
2821 GTGCCACCTG ACGTCTAAGA AACCATTATT ATCATGACAT TAACCTATAA AAATAGGCGT
2881 ATCACGAGGC CCTTTCGTCT TCAAGCTGCC TCGCGCGTTT CGGTGATGAC GGTGAAAACC
2941 TCTGACACAT GCAGCTCCCG GAGACGGTCA CAGCTTGTCT GTAAGCGGAT GCCGGGAGCA
3001 GACAAGCCCG TCAGGGCGCG TCAGCGGGTG TTGGCGGGTG TCGGGGCGCA GCCATGACCC
3061 AGTCACGTAG CGATAGTTAC TATGCGGCAT CAGAGCAGAT TGTACTGAGA GTGCACCATA
3121 TGCGGTGTGA AATACCGCAC AGATGCGTAA GGAGAAAATA CCGCATCAGG CGCCATTCGC
3181 CATTCAGGCT GCGCAACTGT TGGGAAGGGC GATCGGTGCG GGCCTCTTCG CTATTACGCC
3241 AGCTGGCGAA AGGGGGATGT GCTGCAAGGC GATTAAGTTG GGTAACGCCA GGGTTTTCCC
3301 AGTCACGACG TTGTAAAACG ACGGCCAGTG AATTAGTACT CTAGCTTAAG TAACGCCATT
3361 TTGCAAGGCA TGGAAAATAC ATAACTGAGA ATAGAGAAGT TCAGATCAAG GTTAGGAACA
3421 GAGAGACAGC AGAATATGGG CCAAACAGGA TATCTGTGGT AAGCAGTTCC TGCCCCGGCT
3481 CAGGGCCAAG AACAGTTGGA ACAGCAGAAT ATGGGCCAAA CAGGATATCT GTGGTAAGCA
3541 GTTCCTGCCC CGGCTCAGGG CCAAGAACAG ATGGTCCCCA GATGCGGTCC CGCCCTCAGC
3601 AGTTTCTAGA GAACCATCAG ATGTTTCCAG GGTGCCCCAA GGACCTGAAA TGACCCTGTG
3661 CCTTATTTGA ACTAACCAAT CAGTTCGCTT CTCGCTTCTG TTCGCGCGCT TCTGCTCCCC
3721 GAGCTCAATA AAAGAGCCCA CAACCCCTCA CTCGGCGCGC CAGTCCTCCG ATAGACTGCG
3781 TCGCCCGGGT ACCCGTATTC CCAATAAAGC CTCTTGCTGT TTGCATCCGA ATCGTGGACT
3841 CGCTGATCCT TGGGAGGGTC TCCTCAGATT GATTGACTGC CCACCTCGGG GGTCTTTCAT
3901 TTGGAGGTTC CACCGAGATT TGGAGACCCC TGCCCAGGGA CCACCGACCC CCCCGCCGGG
3961 AGGTAAGCTG GCCAGCGGTC GTTTCGTGTC TGTCTCTGTC TTTGTGCGTG TTTGTGCCGG
4021 CATCTAATGT TTGCGCCTGC GTCTGTACTA GTTGGCTAAC TAGATCTGTA TCTGGCGGTC
4081 CCGCGGAAGA ACTGACGAGT TCGTATTCCC GGCCGCAGCC CCTGGGAGAC GTCCCAGCGG
4141 CCTCGGGGGC CCGTTTTGTG GCCCATTCTG TATCAGTTAA CCTACCCGAG TCGGACTTTT
4201 TGGAGCTCCG CCACTGTCCG AGGGGTACGT GGCTTTGTTG GGGGACGAGA GACAGAGACA
4261 CTTCCCGCCC CCGTCTGAAT TTTTGCTTTC GGTTTTACGC CGAAACCGCG CCGCGCGTCT
4321 TGTCTGCTGC AGCATCGTTC TGTGTTGTCT CTGTCTGACT GTGTTTCTGT ATTTGTCTGA
4381 AAATTAGCTC GACAAAGTTA AGTAATAGTC CCTCTCTCCA AGCTCACTTA CAGGCGGCCG
4441 CCATGCCGCG GGGCTGGACC GCGCTTTGCT TGCTGAGTTT GCTGCCTTCT GGGTTCATGA
4501 GTCTTGACAA CAACGGTACT GCTACCCCAG AGTTACCTAC CCAGGGAACA TTTTCAAATG
4561 TTTCTACAAA TGTATCCTAC CAAGAAACTA CAACACCTAG TACCCTTGGA AGTACCAGCC
4621 TGCACCCTGT GTCTCAACAT GGCAATGAGG CCACAACAAA CATCACAGAA ACGACAGTCA
4681 AATTCACATC TACCTCTGTG ATAACCTCAG TTTATGGAAA CACAAACTCT TCTGTCCAGT
4741 CACAGACCTC TGTAATCAGC ACAGTGTTCA CCACCCCAGC CAACGTTTCA ACTCCAGAGA
4801 CAACCTTGAA GCCTAGCCTG TCACCTGGAA ATGTTTCAGA CCTTTCAACC ACTAGCACTA
4861 GCCTTGCAAC ATCTCCCACT AAACCCTATA CATCATCTTC TCCTATCCTA AGTGACATCA
4921 AGGCAGAAAT CAAATGTTCA GGCATCAGAG AAGTGAAATT GACTCAGGGC ATCTGCCTGG
4981 AGCAAAATAA GACCTCCAGC TGTGCGGAGT TTAAGAAGGA CAGGGGAGAG GGCCTGGCCC
5041 GAGTGCTGTG TGGGGAGGAG CAGGCTGATG CTGATGCTGG GGCCCAGGTA TGCTCCCTGC
5101 TCCTTGCCCA GTCTGAGGTG AGGCCTCAGT GTCTACTGCT GGTCTTGGCC AACAGAACAG
5161 AAATTTCCAG CAAACTCCAA CTTATGAAAA AGCACCAATC TGACCTGAAA AAGCTGGGGA
5221 TCCTAGATTT CACTGAGCAA GATGTTGCAA GCCACCAGAG CTATTCCCAA AAGACCCTGA
5281 TTGCACTGGT CACCTCGGGA GCCCTGCTGG CTGTCTTGGG CATCACTGGC TATTTCCTGA
5341 TGAATCGCCG CAGCTGGAGC CCCACAGGAG AAAGGCTGGA ACTAGAACCA ATGGCTTCGT
5401 ACCCCTGCCA TCAACACGCG TCTGCGTTCG ACCAGGCTGC GCGTTCTCGC GGCCATAGCA
5461 ACCGACGTAC GGCGTTGCGC CCTCGCCGGC AGCAAGAAGC CACGGAAGTC CGCCTGGAGC
5521 AGAAAATGCC CACGCTACTG CGGGTTTATA TAGACGGTCC TCACGGGATG GGGAAAACCA
5581 CCACCACGCA ACTGCTGGTG GCCCTGGGTT CGCGCGACGA TATCGTCTAC GTACCCGAGC
5641 CGATGACTTA CTGGCAGGTG CTGGGGGCTT CCGAGACAAT CGCGAACATC TACACCACAC
5701 AACACCGCCT CGACCAGGGT GAGATATCGG CCGGGGACGC GGCGGTGGTA ATGACAAGCG
5761 CCCAGATAAC AATGGGCATG CCTTATGCCG TGACCGACGC CGTTCTGGCT CCTCATGTCG
5821 GGGGGGAGGC TGGGAGTTCA CATGCCCCGC CCCCGGCCCT CACCCTCATC TTCGACCGCC
5881 ATCCCATCGC CGCCCTCCTG TGCTACCCGG CCGCGCGATA CCTTATGGGC AGCATGACCC
5941 CCCAAGCCGT GCTGGCGTTC GTGGCCCTCA TCCCGCCGAC CTTGCCCGGC ACAAACATCG
6001 TGTTGGGGGC CCTTCCGGAG GACAGACACA TCGACCGCCT GGCCAAACGC CAGCGCCCCG
6061 GCGAGCGGCT TGACCTGGCT ATGCTGGCCG CGATTCGCCG CGTTTACGGG CTGCTTGCCA
6121 ATACGGTGCG GTATCTGCAG GGCGGCGGGT CGTGGTGGGA GGATTGGGGA CAGCTTTCGG
6181 GGACGGCCGT GCCGCCCCAG GGTGCCGAGC CCCAGAGCAA CGCGGGCCCA CGACCCCATA
6241 TCGGGGACAC GTTATTTACC CTGTTTCGGG CCCCCGAGTT GCTGGCCCCC AACGGCGACC
6301 TGTATAACGT GTTTGCCTGG GCCTTGGACG TCTTGGCCAA ACGCCTCCGT CCCATGCACG
6361 TCTTTATCCT GGATTACGAC CAATCGCCCG CCGGCTGCCG GGACGCCCTG CTGCAACTTA
```

6421 CCTCCGGGAT GGTCCAGACC CACGTCACCA CCCCAGGCTC CATACCGACG ATCTGCGACC
6481 TGGCGCGCAC GTTTGCCCGG GAGATGGGGG AGGCTAACTA GTA

APPENDIX 2: BIORELIANCE TESTSING OF MCB AND CLINICAL BATCH

Summary of investigations undertaken. Further details are supplied in the IMPD and full specifications are available on request.

Tests performed on MCB

Control criteria	Method	Result
Vector titre > 10^5 ip/ml	Flow cytometry for CD34 on tranduced HT1080 cells	Range 1-3x10^5/ml-
Sterility	Direct Incoulation	No growth of bacteria or fungi
Species identity	PCR for species specific isoenzymes	Sample DNA has to be negative for human, simian, hamster and rat sequences and positive for sequences.
Mycoplasma	PCR,Fluorochrome, cultivation	Negative
Detection Inapparent viruses	In-Vivo Assay in suckling mice, adult mice, guinea pigs	None of the viruses tested for detectable.
MAP test	Inoculation of mice and LCMV challenge	None of 16 viruses tested detected.
Test for bovine viruses via coculture with BT cells	Cellular Assay	No CPE, hemadsorption or hemagglutination is detectable.

Test for porcine viruses via coculture with PPK cells	Cellular Assay	No CPE, hemadsorption or hemagglutination detectable.
Test for adventitious viruses via coculture with Vero, MRC and other lines	Cellular Assay	No CPE, hemadsorption or hemagglutination detectable.
Test for replication competent retroviruses (RCR)	PG4 S+L-Assay	No CPE is detectable.
Test for bovine Polyomavirus (BPyV)	qPCR	No BPyV sequences are detectable
Recovery of cells after thawing	Microscopy	Cell proliferation detectable

Testing on Clinical Batch

Test	Test Article	Method	Specification	Results
Test for replication competent retroviruses (RCR)	EOP cells	PG4 S+L- Assay	No CPE is detectable. The positive, inhibition and negative controls have to show the validity of the assay.	Pending: Vector will not be released unless specification met
Titre of the produced supernatant	Vector supernatant	FACS analysis	> 10^5 infectious particles/ml detectable. The positive and negative controls have to show the validity of the assay.	10^5
Transgene functionality	Vector supernatant	MTT assay	Survival of T cells transduced with HSVTK should be <20% at concentrations of GCV at 10µM using the MTT assay, and no viable cells detected on trypan blue staining	Transgene functional.
Sterility of the cells	Vector supernatant	BacTec	No microbial growth detectable	No microbial growth detected.

Test for replication competent retroviruses (RCR)	Vector supernatant	PG4 S+L- Assay	No CPE is detectable. The positive, inhibition and negative controls have to show the validity of the assay.	Pending: Vector will not be released unless specification met
Endotoxin	Vector supernatant	Chromogenic kinetic LAL test (Ph. Eur.)	< 5 EU / ml. The positive, inhibition and negative controls have to show the validity of the assay.	Pass
Test for mycoplasma via indicator cell culture and culture method	Vector supernatant	Ph. Eur.	No mycoplasma is detectable by coculture in solid and liquid media as well as by fluorescence staining of indicator cells. The positive, inhibition and negative controls have to show the validity of the assay.	Pass

APPENDIX 3: T CELL TRANSDUCTION DATA

Day	Procedures	Control
Day 1	1. 1.Ficoll 250ml blood<br>2. Resuspend at $1.2x10^6$/ml in 2 bags<br>3. Culture medium:<br>a. 200ml X-VIVO 10<br>b. 2ml L-glutamine stock<br>c. 10ml human serum<br>d. 20ul IL-2 (1X106 stock)<br>e. 2ml CD3/CD28 beads (108 beads/ml)<br>4. Coat 8 bags with Retronectin (250ul RN 1mg/ml +10ml PBS per bag)	Cell count $240x10^6$<br>Viability 100%
Day 3	1. Thaw 200ml vector<br>2. Remove RN from 4 bags and add 50ml vector<br>3. Spin 4 bags at 22°C, 1000g, 40 min<br>4. Transfer 50ml cell suspension to each bag	
Day 4	1. Thaw 200ml vector<br>2. Remove RN from 4 bags and add 50ml vector<br>3. Spin 4 bags at 22°C,1000g, 40 min<br>4. Volume reduce cell suspension to 50ml and transfer to newly coated bags<br>5. IL2 added to final concentration 100u/ml	Cell count $210x10^6$<br>Viability 100%
Day 6	1. IL2 added to final concentration 100u/ml	
Day 7	1. Assess CD34 expression by flow cytometry<br>2. Remove CD3/CD28 beads using Magsep<br>3. Rest overnight in X-Vivo 10, 5% AB serum, IL2 100u/ml	Transduction efficiency 8%<br><br>Total cells: $778x10^6$
Day 8	1. CliniMacs selection of CD34+ cells<br>2. Rest overnight in X-Vivo 10, 5% AB serum, IL2 100u/ml	
Day 9	1. Flow cytometry for CD34 purity<br>2. Phenotype analysis<br>3. Samples for spectratyping<br>4. Initiate GCV elimination assay<br>5. Cryopreserve.	CD34+ transduced T cells: $18x10^6$<br>Purification:99%

1. Sample transduction efficiency and purity of selection

T cells were analysed for CD34 expression after transduction (left panel) and then purified by CliniMacs selection (right panel). After selection 99% of T cells (R1) expressed CD34.

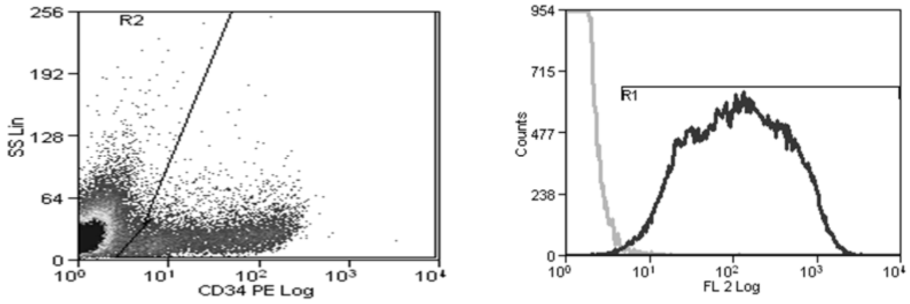

2. T cell phenotype after transduction

T cell phenotype before manipulation (left panel) and after transduction and selection (right panel, gated on $CD34^+$ T cells). The proportion of naive $CD4^+$ $CD45RA^+$ cells is maintained after transduction.

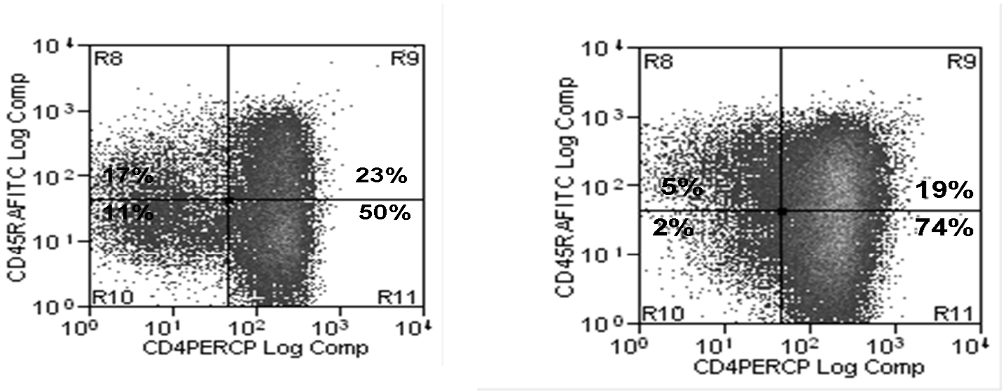

Initial CD4/CD45RAphenotype Final CD4/CD45RAphenotype

3. T cell repertoire analysis

Samples of CD4 and CD8 T cells were separated using magnetic beads before and after transduction, and the T cell receptor Vβ CDR3 region analysed. The data shows preservation of a Gaussian distribution in across the Vβ families, indicating an intact T cell repertoire.

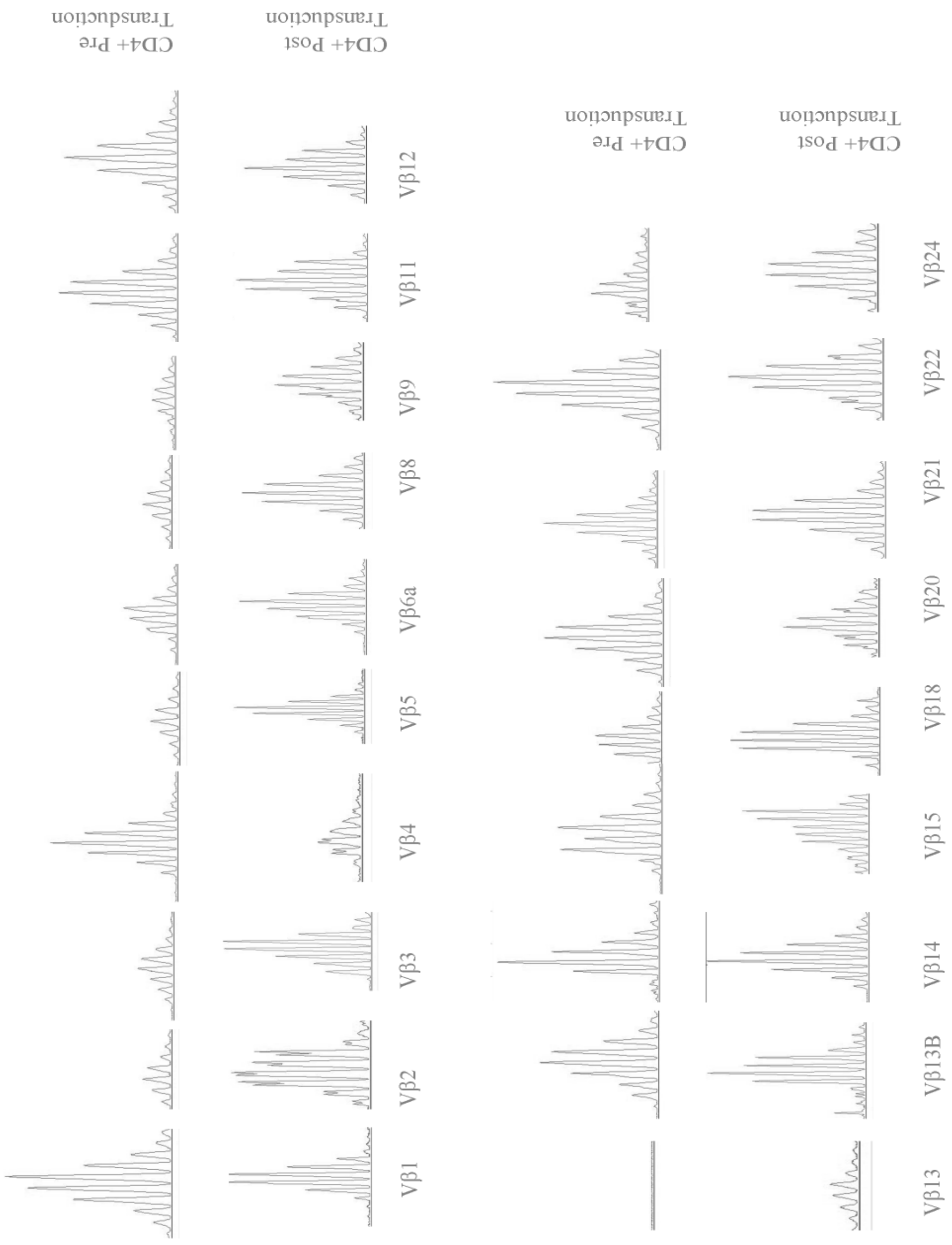

4. T cell alloreactivity and abrogation of alloreactivity after transduction

The alloreactive potential of non transduced (NT) and transduced (CD34+) T cells was assessed in a five day mixed lymphocyte reaction (MLR) against irradiated allogeneic stimulator cells. The proliferation response was measured by uptake of 3H thymidine. The response were abrogated by GCV

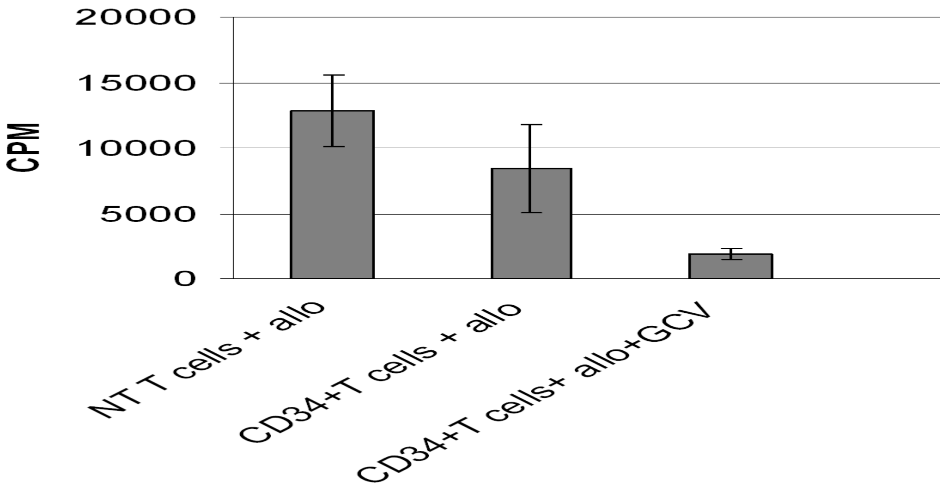

5.T cell elimination after exposure to GCV

Susceptibility of the CD34+ selection T cell fraction to GCV was compared against the CD34- non transduced fraction in a spectrophotometric assay (MTT).

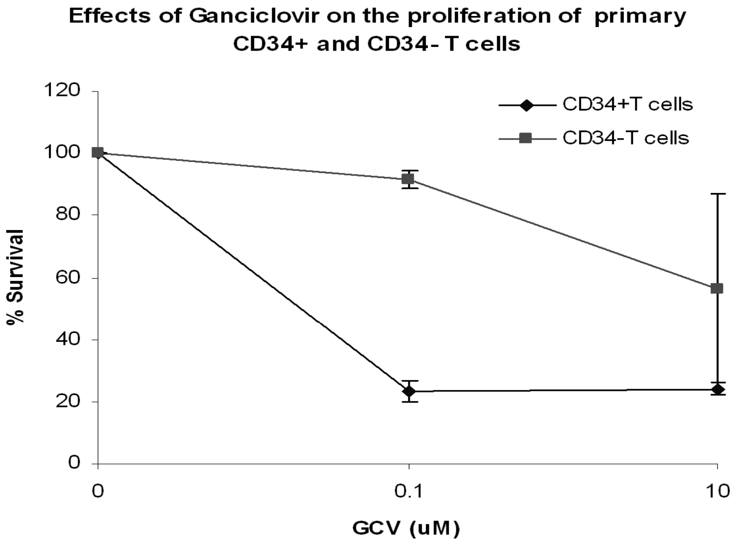

APPENDIX 4: STANDARD TEMPLATE FOR HSCT

HSCT with scHSVTK Gene modified T cell infusion(s)

**PAT NAME:**	**HOSP NO:**	**DOB:**
**WT (kg):**	**HT (cm):**	**SA (m^2):**
**ABO:**	**CMV:**	**EBV:**

**DONOR NAME:**	**HOSP OR ANRC NO:**	**DOB:**
**RELATIONSHIP:**	**WT (kg):**	**SEX:**
**ABO:**	**CMV:**	**EBV:**

<u>Blood products</u>
- All blood products to be irradiated
- All patients on IVIG with CMV negative donor should receive CMV negative products. If donor and/or recipient are CMV positive unscreened products may be used
- RBCS group: (Consider transfusion if Hb < 8.0 g/dl)
- Plts and plasma group: (Consider transfusion if Plt count < 20 x 10^9/l

<u>Infection monitoring</u>
- Weekly CMV, EBV & Adenovirus blood PCR.
- Weekly urine DEAFF for CMV
- Twice weekly NPA for respiratory viruses
- Weekly stool for virus screen and bacterial culture
- Weekly blood culture

TEMPLATE OF INVESTIGATIONS PRIOR TO HSVTK/CD34 T CELL THERAPY

PATIENT NAME: ……………………… **HOSP NO:** ……………………………….

INVESTIGATIONS	*DATE DONE*	*COMMENT*
**Haematology**		
Full blood count		
Coagulation studies		
Blood group		
**Bone marrow lab**		
Engraftment studies		
**Biochemistry**		
U&E's/LFT's		
**Virology**		
CMV PCR, HIV 1&2 PCR, HSV PCR, EBV PCR, HBsAg, Hep C PCR, Adenovirus PCR		all by PCR in Virology
**Microbiology**		
**Clinical Immunology**		
LSS, PHA, IgG, IgA, IgM, IgE		
**Molecular Immunology**		
Immune reconstitution studies, TRECs/T and B spectratyping (pre therapy values)		5 mls EDTA to Molecular Immunology
**PREGNACY TEST (if child bearing age)**		

APPENDIX 5 MONITORING SCHEDULE

	Timepoint (in days) RELATIVE TO DAY OF HSC TRANSPLANTATION												
	**-7 or preconditioning**	**+14**	**+28**	**+42**	**+56**	**+70**	**+84**	**+98**	**+112**	**+140**	**+180**	**+270**	**+360**
FBC + U+Es + LFTs	X	X	X	X	X	X	X	X	X	X	X	X	X
LSS	X	X	X	X	X	X	X	X	X	X	X	X	X
PHA*								X			X		X
IgG IgA IgM **	X								X		X		X
TCR spectratype *	X			X					X		X		X
TRECS *	X								X				X
Detection of Modified T cells<br>• Flow cytometry for CD3+CD34+ T cells<br>• Quantitative PCR for HSVTK if transduced cells <1% on cytometry		X	X		X		X		X		X	X	X
Sample stored for integration analysis								X			X		X
Engraftment studies			X		X		X		X		X	X	X
Sample stored for RCR testing								X			X		X
SCLN	X							X			X		X

LSS - lymphocyte subsets, PHA - phytohaemagglutinin stimulation, Ig – immunoglobulin, TCR - T cell receptor,
RCR - replication competent retrovirus, TREC - TCR excision circles, PCR - polymerase chain reaction, SCLN - save cells in liquid nitrogen

** Assay can only be performed if the lymphocyte count > 0.5 x 10^9/L*

*** Pre-cell therapy assay should be carried out only if not already done at diagnosis*
